# Trends and projections of the global burden of thyroid cancer from 1990 to 2030

**DOI:** 10.7189/jogh.14.04084

**Published:** 2024-05-17

**Authors:** Supei Hu, Xianjiang Wu, Hua Jiang

**Affiliations:** 1 Department of General Practice, Shanghai East Hospital, School of Medicine, Tongji University, Shanghai, China; 2 Research and Education department, Ningbo No.2 Hospital, Ningbo, China; 3 Department of Thyroid Surgery, Ningbo No.2 Hospital, Ningbo, China

## Abstract

**Background:**

We aimed to explore the burden of thyroid cancer worldwide from 1990 to 2019 and to project its future trends from 2020 to 2030.

**Methods:**

Based on annual data on thyroid cancer cases from 1990 to 2019 available in the Global Burden of Disease (GBD) database, we calculated the age-standardised incidence, death, and disability-adjusted life year (DALY) rates for thyroid cancer. We used the estimated annual percentage change (EPAC) to quantify the temporal trends in these age-standardised rates from 1990 to 2019 and applied generalised additive models to project the disease burden from 2020 to 2030.

**Results:**

The global age-standardised incidence rate (ASIR) of thyroid cancer increased from 1990 to 2019, with a higher overall disease burden in women than in men at both study time points. The male-to-female ratios for the ASIR increased from 0.41 in 1990 to 0.51 in 2019, while the ratio for the age-standardised death rate (ASDR) increased from 0.60 to 0.82. The models predicted the United Arab Emirates would have the fastest rising trend in both the ASIR (estimated annual percentage changes (EAPC) = 4.19) and age-standardised DALY rate (EAPC = 4.36) in 2020–30, while Saint Kitts and Nevis will have the fastest rising trend in the ASDR (EAPC = 2.29). Meanwhile, the growth trends for the ASDR and age-standardised DALY rate are projected to increase across countries in this period. A correlation analysis of the global burden of thyroid cancer between 1990–2019 and 2020–30 showed a significant positive correlation between the increase in the ASIR and socio-demographic index (SDI) in low-SDI and low-middle-SDI countries.

**Conclusions:**

The global burden of thyroid cancer is increasing, especially in the female population and in low-middle-SDI regions, underscoring a need to target them for effective prevention, diagnosis, and treatment.

Thyroid cancer has become a significant global health concern [1], with its continually increasing incidence over the past few decades making it one of the most common endocrine system malignancies [2,3]. This has had far-reaching consequences for the overall health and quality of life of patients [4,5]. The disease is characterised by disparities between various demographic groups. For example, women were found to have a higher incidence of thyroid cancer than men, possibly due to gender-based differences such as hormonal influences which may predispose women to a higher risk of acquiring the disease, but also other aspects such as genetic susceptibility, environmental exposure, and certain lifestyle factors [6–8]. Moreover, while thyroid cancer can occur at any age, it is most commonly diagnosed in adults aged 30–60 years, indicating a need for targeted awareness and screening strategies across different age groups [9–11]. The prognosis and treatment outcomes for thyroid cancer are generally favourable, with a generally high survival rate, especially when detected early. This underscores the importance of early detection, effective treatment strategies, and further research into its causes and management to enhance patient survival and improve quality of life.

However, the disease’s mortality rate, although relatively low compared to other cancers, varies across different regions and populations, as does its incidence. This variability may be related to differences genetic backgrounds, environmental conditions, medical resources, and health policies. Therefore, a better understanding of population patterns and the effects of various influencing factors on thyroid cancer could help with improving treatment approaches and reducing the global health burden of thyroid cancer [11].

To address this challenge, we sought to analyse the age-standardised incidence rate (ASIR), age-standardised death rate (ASDR), and age-standardised disability-associated life years (DALY) rate of thyroid cancer worldwide and their estimated annual percentage changes (EAPCs) for the 1990–2019 period based on data from the Global Burden of Disease (GBD) database. Further, to inform future policymaking efforts, we used generalised additive models (GAMs) to predict the global trends in these age-standardised rates for the next decade (2020–30) [12,13].

## METHODS

### Research data

The GBD 2019 is the latest iteration of the GBD surveys designed to assess the burden of incidence, prevalence, mortality, and DALYs for more than 350 diseases, injuries, and risk factors across 204 countries and regions [14,15]. Its up-to-date, transparent database serves as an important source of information for researchers and policymakers worldwide. As our study is based on this publicly available data set, we required no specific ethical approval [16].

We used the socio-demographic index (SDI) to analyse the disease burden by the level of socio-demographic development in different countries and regions. The SDI is a core composite indicator that measures the level of social and economic development of a country or region based on per capita income, average years of schooling, and fertility rates. These regions are then classified by their SDI according to the fifth percentile [17,18]:

− Low-SDI: SDI values from 0 to 0.45, indicating low-income levels, low educational attainment, and high fertility rates;
− Low- and medium-SDI: SDI values from 0.45 to 0.60, suggesting slightly better socio-economic development than the previous category;
− Medium-SDI: SDI values from 0.60 to 0.68, denoting a medium level of socio-economic development;
− Medium-high-SDI: SDI values ranging from 0.68 to 0.80, suggesting higher levels of economic and educational attainment, and lower fertility rates;
− High-SDI: with SDI values from 0.80 to 1, characterised by the highest levels of income and education and the lowest fertility rates, reflecting the highest levels of socioeconomic development.

We extracted thyroid cancer cases based on International Classification of Disease codes (ninth revision: 193–193.9 and 226–226.9, tenth revision: C73–C73.9, D09.3, D09.8, D34–D34.9, and D44.0) [19,20].

### Statistical analysis

We extracted thyroid cancer burden indicators from the GBD data to assess the disease’s global impact. Specifically, we used standardised population age structures and EAPCs to estimate the global ASIR, ASDR, and age-standardised DALY rate trends for thyroid cancer [21,22], after which we combined incidence data and disability weights to calculate the DALY rates.

We calculated age-standardised rates and their associated uncertainty intervals (UIs) using the following standardised formula:



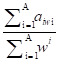



where *ɑi* represents the rate for an age group and *wi* represents the weight of the corresponding age group in the selected reference standardised population (*i* represents the *ith* age group).

We performed 1000 simulations for each incidence, mortality, and DALY rate using the simulation methodology to propagate the 95% UI, with 2.5 and 97.5 set as the lower and upper limits of the 95% UI.

We considered age-standardised rates to have an increasing trend if both the EAPC and the lower limit of its 95% confidence interval (CI) were greater than 0; a decreasing trend if both the EAPC and the upper limit of its 95% CI were less than 0; and a stable trend without significant changes otherwise.

To measure the changing trends in the age-standardised rates of thyroid cancer, we determined their EAPCs and 95% CIs were using the following GAM:

*τ* = β_0_ + *f*_1_(*x*_1_) + *f*_2_(*x*_2_) + … + *f*_n_(*x*_n_) + *∊*

where *τ* is the response variable, i.e. the age-standardised rate for thyroid cancer, β_0_ is the intercept term of the model, *f_i_x_i_* is the additive smoothing function that captures the nonlinear relationship between the independent variable (e.g. time, age, or geographic location) and the response variable, and *ϵ* is the error term which represents unobservable stochastic variation.

GAMs have unique advantages in predicting disease trends as they combine the explanatory nature of linear models with the flexibility of nonlinear models, allowing us to account for complex nonlinear relationships in our projections. They can also be used to explore the possible effects of time on disease burden. We used time as a smoothing term in GAMs to capture trends over time and conducted subgroup analyses for different geographic regions and age groups.

Furthermore, GAMs can capture the dynamics of disease trends through a smoothing function over time. This smoothing function can be a nonlinear trend based on years, which can more accurately reflect the pattern of disease change over time. Using historical data, GAMs can predict the future trends in the thyroid cancer burden while accounting for the continuity of current trends and potential nonlinear changes. When making predictions, we used GAMs to provide an estimate of the uncertainty of the prediction, which is denoted by prediction intervals (CIs).

Lastly, we used linear regression analyses to examine the relationship between the SDI and thyroid cancer rates. Specifically, we used Pearson correlation coefficient to quantify the strength and direction of the association.

## RESULTS

### Global trend forecast of the overall disease burden of thyroid cancer, 1990–2020 and 2020–30

**Figure 1:**
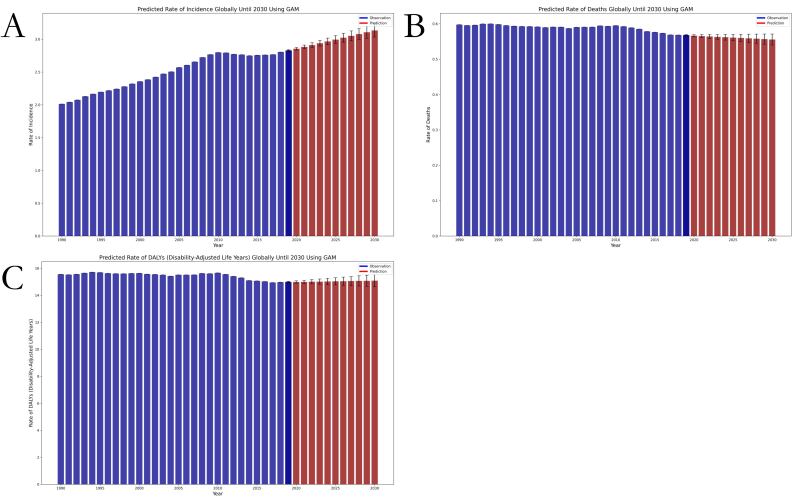
Trends in global thyroid cancer burden from 1990 to 2019 and projected rates from 2020 to 2030 using GAMs modelling. **Panel A.** ASIR. **Panel B.** ASDR. **Panel C.** Age-standardised DALY rate.

From 1990 to 2019, the global ASIR of thyroid cancer increased from 2.01 (95% CI = 1.90, 2.12) to 2.83 (95% CI = 2.56, 3.06) per 100 000 population; the global ASDR decreased from 0.60 (95% CI = 0.56, 0.66) to 0.57 (95% CI = 0.51, 0.61) per 100 000 population; and the global age-standardised DALY rate decreased from 15.55 per 100 000 population (95% CI = 14.40, 17.02) to 14.98 (95% CI = 13.55, 16.14) per 100 000 population. This indicates that the incidence of the disease increased annually, while the burden of death and its impact on the population’s life expectancy loss declined (**Table 1**; Table S1 in the **Online Supplementary Document**). The GAM model estimated that the overall ASIR will rise rapidly from 2020 to 2030, the ASDR will slowly decline, and the age-standardised DALY rate will gradually increase (**Figure 1**).

**Table 1:** The age-standardised rate of thyroid cancer incidences, deaths, and DALYs in 1990 and 2019 and its trend between 1990–2019

	1990			2019			1990–2019 EAPC		
**Location**	**ASIR per 100 000 population (95%UI)**	**Age-standardised DALY rate per 100 000 population (95%UI)**	**ASDR per 100 000 population (95%UI)**	**ASIR per 100 000 population (95%UI)**	**Age-standardised DALY rate per 100 000 population (95%UI)**	**ASDR per 100 000 population (95%UI)**	**ASIR per 100 000 population (95% CI)**	**Age-standardised DALY rate per 100 000 population (95% CI)**	**ASDR per 100 000 population (95% CI)**
Global	2.01 (1.90, 2.12)	15.55 (14.40, 17.02)	0.60 (0.56, 0.66)	2.83 (2.56, 3.06)	14.98 (13.55, 16.14)	0.57 (0.51, 0.61)	1.25 (1.12, 1.38)	−0.14 (−0.18, −0.09)	−0.15 (−0.19, −0.11)
SDI									
*High-middle SDI*	2.28 (2.13, 2.40)	15.45 (14.37, 16.34)	0.60 (0.56, 0.63)	3.06 (2.76, 3.38)	12.35 (11.27, 13.41)	0.47 (0.43, 0.51)	1.05 (0.94, 1.16)	−0.91 (−0.99, −0.83)	−0.90 (−0.97, −0.82)
*High SDI*	3.40 (3.28, 3.50)	13.87 (13.15, 14.67)	0.56 (0.52, 0.58)	4.59 (4.17, 5.03)	12.29 (11.13, 13.31)	0.47 (0.42, 0.50)	1.21 (0.89, 1.52)	−0.37 (−0.51, −0.22)	−0.55 (−0.63, −0.48)
*Low-middle SDI*	1.13 (0.97, 1.33)	16.08 (13.93, 19.28)	0.59 (0.52, 0.70)	1.91 (1.66, 2.13)	17.75 (15.52, 19.65)	0.66 (0.58, 0.73)	1.80 (1.74, 1.85)	0.32 (0.24, 0.40)	0.40 (0.32, 0.48)
*Low SDI*	1.23 (0.98, 1.57)	21.13 (16.86, 26.82)	0.73 (0.61, 0.90)	1.63 (1.34, 1.90)	19.57 (16.03, 22.66)	0.72 (0.59, 0.84)	0.95 (0.90, 0.99)	−0.34 (−0.38, −0.30)	−0.07 (−0.10, −0.03)
*Middle SDI*	1.31 (1.20, 1.51)	14.33 (13.23, 16.91)	0.56 (0.51, 0.68)	2.49 (2.19, 2.77)	15.02 (13.35, 16.61)	0.59 (0.53, 0.66)	2.36 (2.27, 2.45)	0.30 (0.23, 0.38)	0.39 (0.30, 0.48)
Region									
*Andean Latin America*	1.76 (1.53, 2.12)	23.71 (20.70, 28.31)	0.95 (0.83, 1.15)	3.63 (2.79, 4.54)	26.74 (20.77, 32.71)	1.13 (0.86, 1.38)	2.68 (2.42, 2.94)	0.54 (0.39, 0.70)	0.81 (0.64, 0.97)
*Australasia*	2.31 (2.13, 2.52)	10.98 (10.27, 11.91)	0.43 (0.40, 0.45)	4.44 (3.47, 5.72)	12.94 (10.98, 14.56)	0.48 (0.40, 0.51)	2.72 (2.36, 3.08)	0.96 (0.77, 1.15)	0.77 (0.59, 0.95)
*Caribbean*	1.70 (1.57, 1.82)	15.60 (13.97, 17.16)	0.59 (0.53, 0.64)	2.43 (2.04, 2.89)	16.77 (13.87, 19.77)	0.62 (0.52, 0.72)	1.42 (1.28, 1.55)	0.45 (0.24, 0.67)	0.44 (0.22, 0.65)
*Central Asia*	1.44 (1.27, 1.67)	13.23 (11.44, 15.38)	0.46 (0.41, 0.54)	1.69 (1.51, 1.89)	11.46 (10.27, 12.79)	0.45 (0.40, 0.49)	0.42 (0.10, 0.74)	−0.88 (−1.12, −0.64)	−0.38 (−0.60, −0.15)
*Central Europe*	3.04 (2.77, 3.16)	21.29 (18.56, 22.17)	0.80 (0.72, 0.83)	3.09 (2.67, 3.57)	12.73 (11.04, 14.75)	0.48 (0.42, 0.56)	−0.17 (−0.26, −0.07)	−2.08 (−2.29, −1.88)	−2.02 (−2.21, −1.83)
*Central Latin America*	1.80 (1.69, 1.87)	20.24 (18.80, 21.00)	0.84 (0.77, 0.87)	2.90 (2.49, 3.40)	20.04 (17.11, 23.15)	0.82 (0.71, 0.94)	1.57 (1.49, 1.66)	−0.08 (−0.22, 0.06)	−0.09 (−0.22, 0.03)
*Central sub-Saharan Africa*	0.72 (0.51, 0.98)	12.69 (9.23, 17.00)	0.51 (0.36, 0.69)	0.80 (0.54, 1.14)	11.63 (8.08, 16.52)	0.49 (0.33, 0.71)	0.34 (0.27, 0.41)	−0.32 (−0.35, −0.30)	−0.14 (−0.16, −0.12)
*East Asia*	1.07 (0.92, 1.27)	11.07 (9.57, 13.32)	0.43 (0.38, 0.53)	2.11 (1.77, 2.54)	9.86 (8.30, 11.35)	0.40 (0.33, 0.46)	2.59 (2.45, 2.73)	−0.22 (−0.32, −0.12)	0.04 (−0.10, 0.17)
*Eastern Europe*	2.28 (2.15, 2.56)	13.56 (12.68, 15.18)	0.51 (0.48, 0.56)	4.25 (3.70, 4.93)	15.82 (14.08, 17.81)	0.55 (0.49, 0.62)	2.44 (2.16, 2.71)	0.26 (−0.05, 0.58)	0.04 (−0.27, 0.34)
*Eastern sub-Saharan Africa*	1.94 (1.41, 2.65)	34.72 (24.99, 47.12)	1.16 (0.89, 1.50)	2.19 (1.72, 2.72)	27.78 (21.85, 34.10)	1.04 (0.80, 1.26)	0.30 (0.15, 0.45)	−0.90 (−1.00, −0.79)	−0.41 (−0.50, −0.32)
*High-income Asia Pacific*	3.32 (3.13, 3.75)	14.06 (13.16, 16.49)	0.63 (0.58, 0.72)	4.98 (4.19, 5.79)	12.46 (10.69, 13.99)	0.53 (0.43, 0.58)	2.16 (1.35,2.98)	−0.05 (−0.55, 0.46)	−0.45 (−0.76, −0.15)
*High-income North America*	3.96 (3.82, 4.08)	11.05 (10.27, 11.88)	0.39 (0.37, 0.41)	5.40 (4.64, 6.27)	12.23 (11.10, 13.34)	0.43 (0.40, 0.45)	1.04 (0.85, 1.24)	0.33 (0.22, 0.43)	0.35 (0.27, 0.43)
*North Africa and Middle East*	1.70 (1.44, 2.00)	14.42 (11.80, 17.96)	0.54 (0.44, 0.74)	3.46 (2.89, 3.96)	14.88 (12.81, 17.09)	0.54 (0.47, 0.66)	2.62 (2.52, 2.71)	0.18 (0.08, 0.29)	0.13 (0.02, 0.24)
*Oceania*	1.45 (1.15, 1.86)	18.15 (14.67, 23.81)	0.74 (0.60, 0.97)	1.80 (1.30, 2.40)	19.04 (14.17, 25.95)	0.78 (0.59, 1.04)	0.68 (0.57, 0.78)	0.18 (0.11, 0.25)	0.23 (0.19, 0.28)
*South Asia*	1.05 (0.90, 1.32)	15.89 (13.63, 20.27)	0.57 (0.49, 0.72)	1.90 (1.61, 2.19)	18.33 (15.98, 20.88)	0.65 (0.56, 0.74)	2.06 (1.94, 2.18)	0.46 (0.30, 0.61)	0.42 (0.25, 0.58)
*Southeast Asia*	2.25 (1.86, 2.55)	25.14 (21.35, 28.51)	0.98 (0.86, 1.12)	3.72 (3.01, 4.32)	25.34 (21.37, 28.88)	1.02 (0.88, 1.15)	1.76 (1.70, 1.82)	0.07 (0.01, 0.12)	0.21 (0.14, 0.28)
*Southern Latin America*	2.04 (1.88, 2.18)	18.60 (16.84, 19.58)	0.75 (0.68, 0.79)	2.58 (2.00, 3.31)	14.25 (13.18, 15.48)	0.58 (0.53, 0.62)	0.67 (0.53, 0.82)	−1.03 (−1.23, −0.82)	−0.99 (−1.17, −0.81)
*Southern sub-Saharan Africa*	1.00 (0.85, 1.10)	11.81 (10.28, 13.08)	0.45 (0.38, 0.51)	1.13 (0.97, 1.30)	11.96 (10.11, 13.61)	0.48 (0.40, 0.54)	0.43 (0.34, 0.53)	0.19 (−0.05, 0.44)	0.29 (0.02, 0.56)
*Tropical Latin America*	1.53 (1.46, 1.61)	16.74 (16.04, 17.61)	0.69 (0.65, 0.72)	1.82 (1.71, 2.02)	12.82 (12.02, 14.57)	0.52 (0.48, 0.58)	0.67 (0.40, 0.94)	−0.82 (−0.96, −0.69)	−0.81 (−0.92, −0.71)
*Western Europe*	3.63 (3.41, 3.77)	16.53 (15.27, 17.32)	0.67 (0.63, 0.70)	3.93 (3.40, 4.50)	11.66 (10.59, 12.79)	0.46 (0.41, 0.48)	0.17 (−0.04, 0.37)	−1.32 (−1.37, −1.27)	−1.49 (−1.55, −1.43)
*Western sub-Saharan Africa*	0.36 (0.28, 0.42)	5.89 (4.62, 6.89)	0.24 (0.18, 0.28)	0.43 (0.34, 0.52)	5.67 (4.43, 6.70)	0.23 (0.18, 0.27)	0.69 (0.64, 0.75)	−0.13 (−0.16, −0.10)	−0.06 (−0.10, −0.03)

ASDR − age-standardised death rate, ASIR − age-standardised incidence rate, CI – confidence interval, DALY − disability-adjusted life years, EAPC – estimated annual percentage changes, SDI − socio-demographic index, UI – uncertainty interval

### Projections of the global distribution of thyroid cancer burden by gender, 1990–2020 and 2020–30

Changes in the global burden of thyroid cancer from 1990 to 2019 differed markedly between genders and were significantly higher in women than in men in both 1990 and 2019. However, this gap narrowed over the 1990–2019 period in terms of the age-standardised rate ratio. Specifically, the male-to-female ratio for the ASIR increased from 0.41 in 1990 to 0.51 in 2019; the ratio for the ASDR increased from 0.60 in 1990 to 0.82 in 2019; and ratio for the age-standardised DALY rate increased from 0.58 in 1990 to 0.76 in 2019 (**Table 1**).

**Figure 2:**
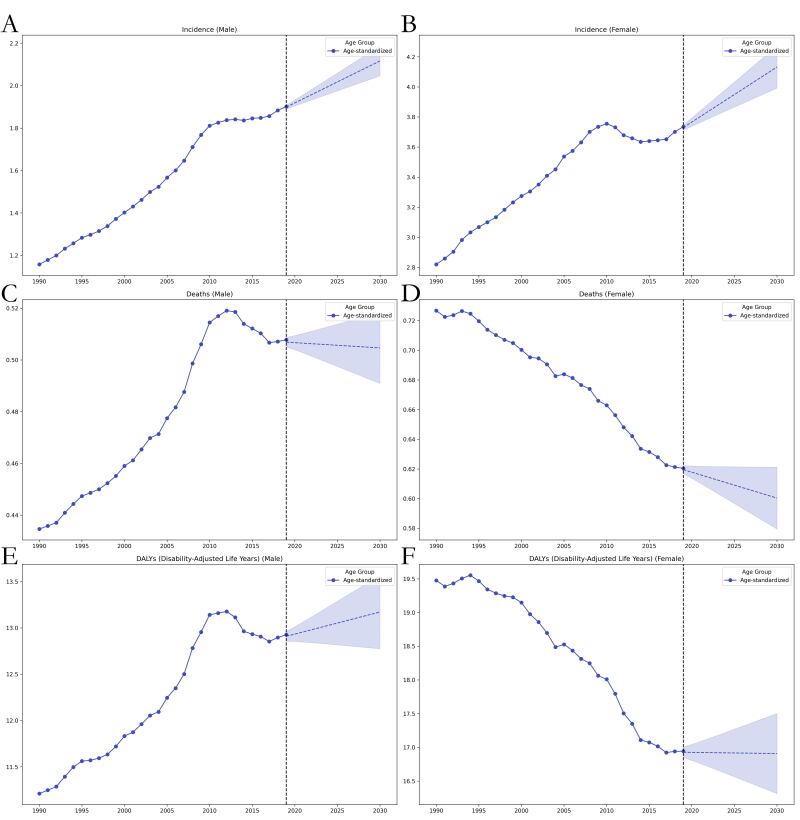
Trends in global thyroid cancer burden from 1990 to 2019 and projected rates from 2020 to 2030 using GAMs modelling, stratified by genders. **Panel A.** ASIR in men. **Panel B.** ASIR in women. **Panel C.** ASDR in men. **Panel D.** ASDR in women. **Panel E.** Age-standardized DALY rate in men. **Panel F.** Age-standardised DALY rate in women.

We observed a general upward trend in ASIR in both men and women in 1990–2019, which our model predicted would continue in 2020–30 due to an increase in the risk of disease. In contrast, although the ASDR showed a general upward trend in men in 1990–2019, the rate began to decrease after 2010; thus, our model predicted that the ASDR in both men and women will decrease in 2020–30. Similarly, the age-standardised DALY rate showed a general upward trend from 1990 to 2019, but began to decline in men post-2010; our model further projects that it will continue this decreasing trend in both men and women in 2020–30. Interestingly, although the 1990–2019 trend of the age-standardised DALY rate in both men and women was similar to that of the ASDR, GAM projections indicated that the loss of life expectancy for men will increase more rapidly in 2020–30, while that in women will remain stable (**Figure 2**).

### Projections of the global distribution of thyroid cancer burden by age, 1990–2020 and 2020–30

The age distribution of the global burden of thyroid cancer between 1990 and 2019 varied considerably. We included people aged ≥10 years in the study and divided the populations into five-year age groups, resulting in a total of 18 age groups. The global death rate for thyroid cancer from 1990 to 2019 showed a gradually increasing trend from the lower to the higher age groups, and the GAM fitting predicted a stable trend in 2020–30 for all of the age groups except for the 90–94 and ≥95-year-old groups, for which the model predicted a decreasing trend.

The distribution of the incidence rate and DALY rate across age groups was predicted to be more complex. First, regarding the global incidence rates of thyroid cancer in 1990–2019, we noted increasing trends with older age in the age subgroups between 10 and 79 years and in the ≥95-year-old age group. While the incidence rates in the 80–84- and 85–89-year-old age groups were comparable to those in the 65–69- and 70–74-year-old age groups, the 90–94-year-old age group had a lower incidence rate, almost comparable to that in the 50–54-year-old age group. Compared with other age groups, we found a greater overall decline in the incidence rate in the 90–94-year-old age group and a moderate one in the 80–89-year-old age group. Our incidence rate projections for 2020–30 showed that the incidence rates in almost all age groups will show an upward trend, except for the ≥80-year-old age groups, which will show a downward trend.

**Figure 3:**
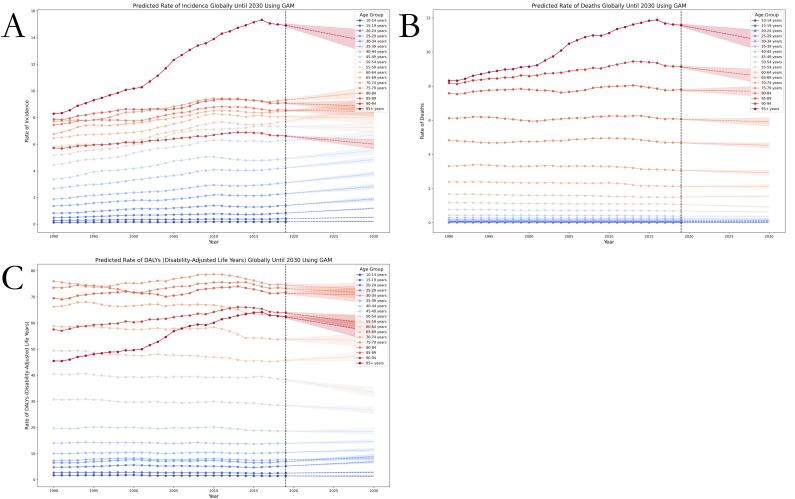
Trends in global thyroid cancer burden from 1990 to 2019 and projected rates from 2020 to 2030 using GAMs modelling, stratified by age group. One age group every five years, with a total of 18 subgroups. **Panel A.** ASIR. **Panel B.** ASDR. **Panel C.** Age-standardised DALY rate.

The trends in the global DALY rate of thyroid cancer for 1990–2019 varied across the age groups, with the 14 groups between 10 and 79 years of age having a trend that increased with age and those in the four ≥80-year-old age groups a trend that that decreased with age. These trends are projected to continue in 2020–30 in most of the age groups. According to our projections for 2020–30, the four ≥80-year-old age groups will have a decreasing trend in the DALY rates with age; however, the trend in the 50–54- and 55–59-year-old age groups is expected to remain identical to that in 1990–2019. The DALY rates in the remaining age groups are projected show an upward trend (**Figure 3**).

### Projections of the global distribution of thyroid cancer burden by country, 1990–2020 and 2020–30

We predicted the disease burden of thyroid cancer for 204 countries in 2020–30 and found that the EAPCs of all disease burden indicators would generally be positive, while the overall disease burden will have an upward trend in all except a few countries. In particular, our model predicted downward trends in the EAPC of the ASIR for 14 countries, in the EAPC of the ASDR for 23 countries, and in the EAPC of the age-standardised DALY rates for 29 countries.

The United Arab Emirates were predicted to have the fastest growing ASIR in 2020–30 (EAPC = 4.19; 95% CI = 4.00, 4.37), followed by Bahrain (EAPC = 3.67; 95% CI = 3.53, 3.82) and Kuwait (EAPC = 3.58; 95% CI = 3.45, 3.72). Saint Kitts and Nevis was predicted to have the fastest growing ASDR between 2020 and 2030 (EAPC = 2.29; 95% CI = 2.23, 2.35), followed by the Bolivarian Republic of Venezuela (EAPC = 2.06; 95% CI = 2.01, 2.10) and the United Arab Emirates (EAPC = 1.99; 95% CI = 1.95, 2.03). The United Arab Emirates were also predicted to have the fastest growing age-standardised DALY rate in 2020–30 (EAPC = 4.36; 95% CI = 4.16, 4.56), followed by Bahrain (EAPC = 3.81; 95% CI = 3.65, 3.96) and Saint Kitts and Nevis (EAPC = 3.81; 95% CI = 3.66, 3.97) (**Table 2**, **Table 3**; Table S2 in the **Online Supplementary Document**).

**Table 2:** The top 10 countries with the largest increase of age-standardised incidence rate, death rate, and disability-adjusted life years rate of thyroid cancer between 2020 and 2030

Order	Countries	EAPC of ASIR, n (95% CI)	Countries	EAPC of ASDR, n (95% CI)	Countries	EAPC of age-standardised DALY rate, n (95% CI)
1	United Arab Emirates	4.19 (4.00, 4.37)	Saint Kitts and Nevis	2.29 (2.23, 2.35)	United Arab Emirates	4.36 (4.16, 4.56)
2	Bahrain	3.67 (3.53, 3.82)	Venezuela	2.06 (2.01, 2.10)	Bahrain	3.81 (3.65, 3.96)
3	Kuwait	3.58 (3.45, 3.72)	United Arab Emirates	1.99 (1.95, 2.03)	Saint Kitts and Nevis	3.81 (3.66, 3.97)
4	Saudi Arabia	3.44 (3.31, 3.56)	Northern Mariana Islands	1.90 (1.86, 1.93)	Cabo Verde	3.73 (3.58, 3.87)
5	Malaysia	3.15 (3.04, 3.25)	Palau	1.66 (1.63, 1.69)	Venezuela (Bolivarian Republic of)	3.63 (3.50, 3.77)
6	Saint Kitts and Nevis	3.11 (3.01, 3.21)	Thailand	1.57 (1.54, 1.60)	Kuwait	3.33 (3.21, 3.45)
7	Egypt	2.91 (2.82, 3.00)	Greece	1.48 (1.45, 1.50)	Northern Mariana Islands	2.72 (2.64, 2.80)
8	Palestine	2.91 (2.82, 3.00)	Guam	1.32 (1.30, 1.33)	Malaysia	2.58 (2.51, 2.65)
9	Maldives	2.86 (2.77, 2.95)	Malaysia	1.30 (1.28, 1.32)	Thailand	2.53 (2.46, 2.60)
10	Iran	2.62 (2.54, 2.69)	Saint Lucia	1.30 (1.28, 1.32)	Jordan	2.52 (2.45, 2.58)

ASDR − age-standardized death rate, ASIR − age-standardised incidence rate, CI – confidence interval, DALY − disability-adjusted life years, EAPC – estimated annual percentage changes

**Table 3:** EAPCs and their confidence intervals for different rates from 1990 to 2019 and from 2020 to 2030, stratified by SDI regions

	From 1990 to 2019			From 2020 to 2030		
**Location**	**DALYs (95% CI)**	**Deaths (95% CI)**	**Incidence (95% CI)**	**DALYs (95% CI)**	**Deaths (95% CI)**	**Incidence (95% CI)**
Global	0.87 (0.39, 1.36)	0.38 (0.21, 0.56)	1.54 (0.95, 2.14)	1.11 (1.10, 1.13)	0.54 (0.54, 0.55)	1.36 (1.34, 1.38)
High SDI	0.68 (0.36, 1.00)	0.42 (0.21, 0.63)	1.71 (1.14, 2.27)	0.90 (0.89, 0.90)	0.75 (0.74, 0.75)	0.40 (0.40, 0.40)
High-middle SDI	0.38 (−0.03, 0.78)	0.26 (0.07, 0.44)	1.66 (0.97, 2.34)	0.98 (0.97, 0.99)	0.54 (0.54, 0.55)	1.37 (1.35, 1.39)
Low SDI	−0.28 (−1.03, 0.48)	−0.04 (−0.22, 0.14)	0.48 (−0.03, 1.00)	0.79 (0.78, 0.79)	0.27 (0.26, 0.27)	1.39 (1.37, 1.41)
Low-middle SDI	1.21 (0.59, 1.84)	0.46 (0.29, 0.63)	1.41 (0.85, 1.96)	1.42 (1.40, 1.44)	0.60 (0.60, 0.60)	2.13 (2.08, 2.17)
Middle SDI	1.77 (1.29, 2.24)	0.68 (0.53, 0.83)	2.31 (1.73, 2.88)	1.40 (1.38, 1.42)	0.66 (0.66, 0.67)	2.24 (2.19, 2.29)

CI – confidence interval, DALY − disability-adjusted life years, SDI – socio-demographic index

Notably, the United Arab Emirates, Bahrain, and Kuwait are projected to experience the fastest increases in ASIR, highlighting regional variations. Similarly, Saint Kitts and Nevis, the Bolivarian Republic of Venezuela, and the United Arab Emirates are expected to lead in ASDR growth. This underscores the influence of regional factors such as health care access, public health policies, and socioeconomic conditions on thyroid cancer trends.

### Projections of the global distribution of thyroid cancer burden by the SDI level, 2020–30

We projected the relationship between the disease burden and SDI from 1990 to 2019 using GAMs. Globally, the EAPC in the ASIR is expected to decrease from 1.54 (95% CI = 0.95, 2.14) in 1990–2019 to 1.36 (95% CI = 1.34, 1.38) in 2020–30, indicating a slower growth trend in the future. The EAPCs of both the ASDR and age-standardised DALY rate were projected to increase from 0.38 (95% CI = 0.21, 0.56) in 1990–2019 to 0.54 (95% CI = 0.54, 0.55) in 2020–30 and from 0.87 (95% CI = 0.39, 1.36) in 1990–2019 to 1.11 (95% CI = 1.10, 1.13) in 2020–30, respectively. These results suggest that the disease burden in terms of deaths and DALYs will be lower than the ASIR, with the ASIR remaining the most rapidly growing disease burden indicator overall (**Table 3**).

**Figure 4:**
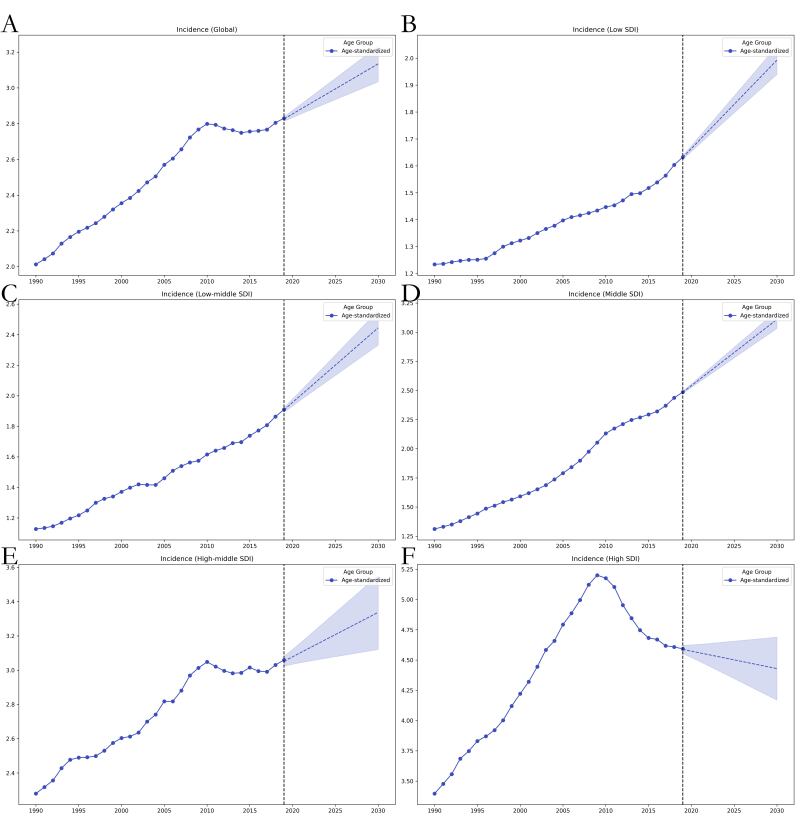
Trends in global ASIR burden for thyroid cancer from 1990 to 2019 and projected ASIR from 2020 to 2030 using the GAMs model, stratified by SDI region. **Panel A.** Global. **Panel B.** Low SDI. **Panel C.** Low-middle SDI. **Panel D.** Middle SDI. **Panel E.** High-middle SDI. **Panel F.** High SDI. ASIR – age-standardised incidence rate.

**Figure 5:**
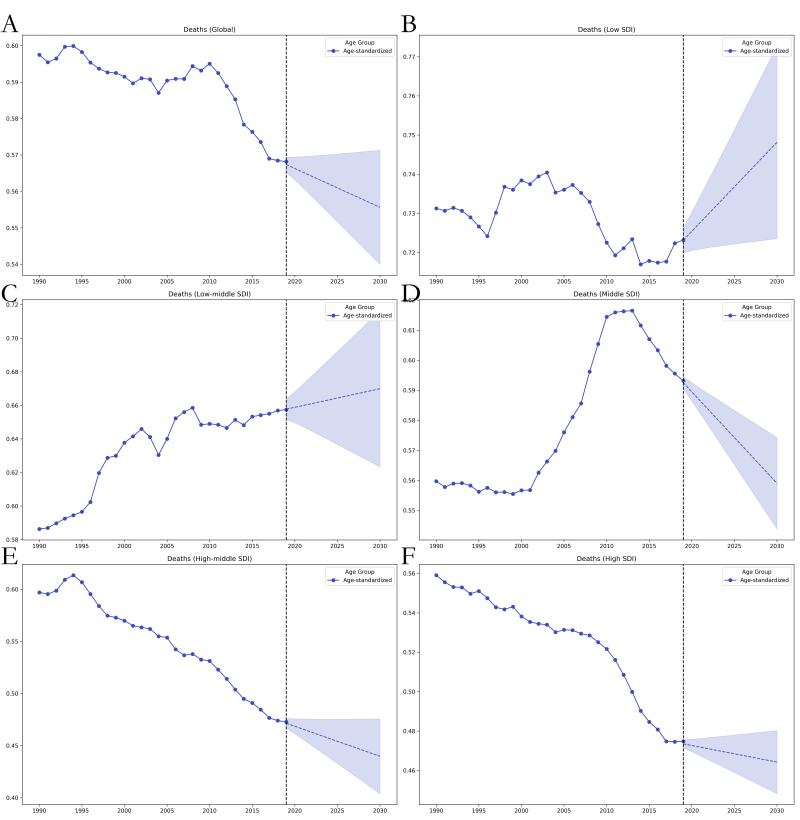
Trends in global ASDR burden for thyroid cancer from 1990 to 2019 and projected ASIR from 2020 to 2030 using the GAMs model, stratified by SDI region. **Panel A.** Global. **Panel B.** Low SDI. **Panel C.** Low-middle SDI. **Panel D.** Middle SDI. **Panel E.** High-middle SDI. **Panel F.** High SDI. ASIR – age-standardised incidence rate.

**Figure 6:**
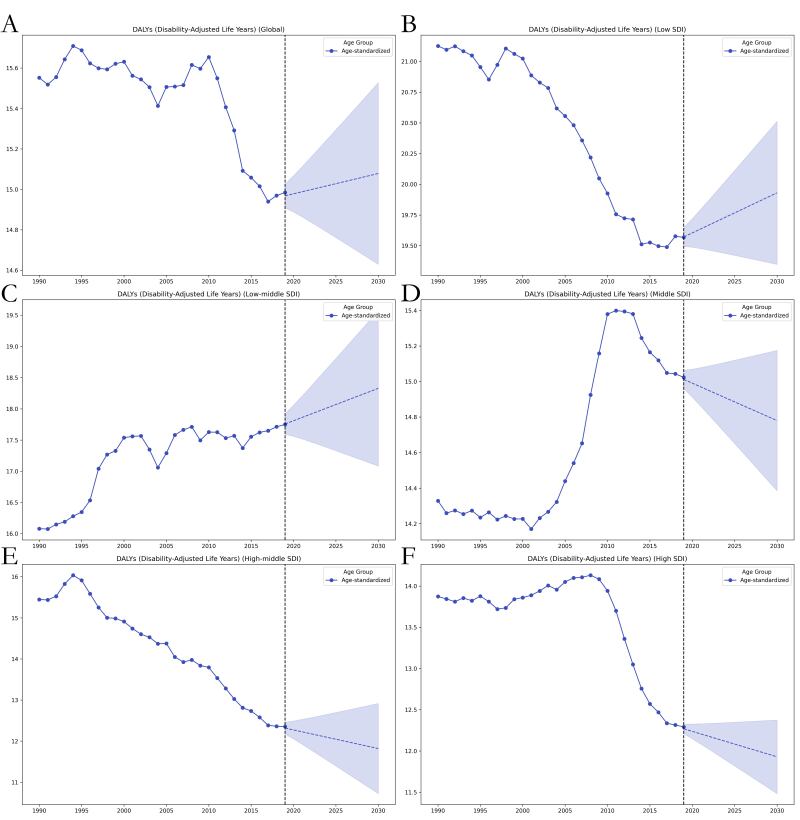
Trends in global age-standardised DALY rate burden for thyroid cancer from 1990 to 2019 and projected ASIR from 2020 to 2030 using the GAMs model, stratified by SDI region. **Panel A.** Global. **Panel B.** Low SDI. **Panel C.** Low-middle SDI. **Panel D.** Middle SDI. **Panel E.** High-middle SDI. **Panel F.** High SDI. ASIR – age-standardised incidence rate.

The GAMs predicted that the ASIR of thyroid cancer from 2020 to 2030 will increase in all except for high-SDI regions, where they will show a decreasing trend. In line with the global trend, the ASDR will show a decreasing trend in middle-SDI and high-SDI regions but an increasing trend in low-SDI and low-middle-SDI regions. In contrast, projections of the age-standardised DALY rate for 2020–2030 showed that the trend would be consistent with the global trend; that is, the rate would trend upward in low-SDI and low-middle-SDI regions and downward in the other SDI regions. Notably, the low-middle- and low-SDI regions had the highest ASDRs and age-standardised DALY rates over the entire 1990–2030 period, whereby in contrast, they had the lowest ASIRs for the same time period (**Figure 4**, **Figure 5**, **Figure 6**, **Table 3**).

### Correlation analysis of the global burden of thyroid cancer between 1990–2019 and 2020–30

**Figure 7:**
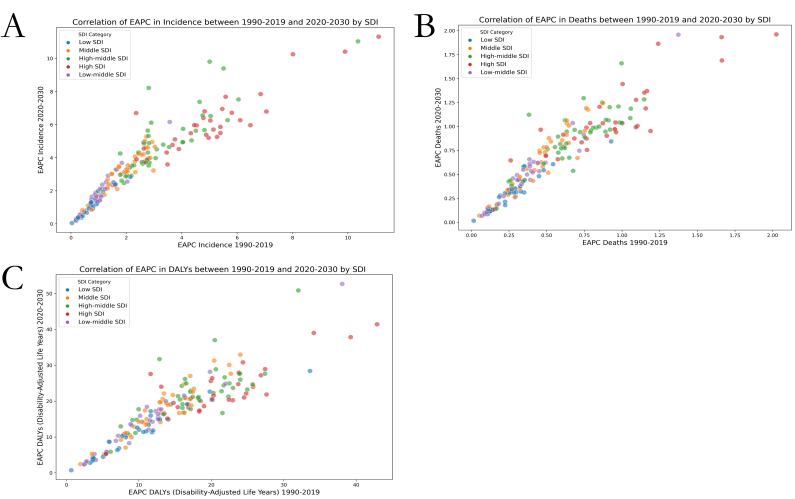
Correlation between EAPC from 1990 to 2019 and EAPC from 2020 to 2030 projected using GAMs modelling, by SDI region. **Panel A.** ASIR. **Panel B.** ASDR. **Panel C.** Age-standardised DALY rate.

We fitted GAMs to predict the global burden of thyroid cancer from 2020 to 2030 as a function of the SDI level and analysed its correlation with the global burden of thyroid cancer from 1990 to 2019. We found no significant correlation of SDI with the EAPCs in the ASIR, ASDR, and age-standardised DALY rate between the two time spans in high- and middle-SDI countries, yet detected a significant positive correlation between SDI and the EAPC in the ASIR in low-SDI and low-middle-SDI countries (low SDI: Correlation coefficient = 0.56, *P* < 0.01; low-middle SDI: Correlation coefficient = 0.61, *P* < 0.01). This indicates that the incidence of disease may increase with socioeconomic development in countries with low SDI. There was also a significant positive correlation between the increase in incidence and SDI in high-middle-SDI countries (correlation coefficient = 0.41, *P* < 0.01), indicating an increase in the incidence of thyroid cancer with SDI in these countries (**Figure 7****,** **Table 4**).

**Table 4:** Correlation between EAPC from 1990 to 2019 and EAPC from 2020 to 2030 projected using GAMs modelling, stratified by SDI region

	EAPC in DALYs		EAPC in deaths		EAPC in incidence	
**Groups**	**Correlation coefficient**	*P*-value*	**Correlation coefficient**	*P*-value*	**Correlation coefficient**	***P*-value**
High SDI	0.280245985	0.114185425	0.303011134	0.086508443	0.221423358	0.215576545
High-middle SDI	0.242662121	0.11248444	0.181241022	0.239040421	0.414584494	0.005141886
Low SDI	0.228859155	0.223804183	0.18313796	0.332699412	0.562948633	0.001200969
Low-middle SDI	0.12343822	0.486737522	0.326515542	0.059469394	0.617624254	9.96E-05
Middle SDI	−0.156404592	0.348386348	0.033130323	0.843466961	0.150735113	0.366350622

DALY − disability-adjusted life years, EAPC – estimated annual percentage changes, SDI – socio-demographic index

*Paired-sample *t*-test.

## DISCUSSION

This is the latest study on the global burden of thyroid cancer based on data from the GBD 2019 study. We found that the incidence of thyroid cancer increased annually from 1990 to 2019, while the mortality rate and the impact of the disease on the population’s life expectancy loss (DALY rate) decreased. Projections for 2020–30 showed an upward trend in the overall global burden of thyroid cancer, with an increase in the EAPCs of both the ASIR and age-standardised DALY rate. Projections for 2020–30 indicate not only an upward trend in the overall global burden of thyroid cancer, but also an opportunity for public health systems to intensify focus on early detection and preventive strategies, particularly in high-incidence areas. This could significantly improve health care quality and patient outcomes, as reflected in the anticipated changes in the EAPCs of both the ASIR and age-standardised DALY rate.

There are several possible explanations for the rising trend in the disease burden of thyroid cancer. First, improved diagnostic techniques and increased health awareness may have led to more cases being diagnosed, thus increasing the disease burden statistics [23,24]. Second, although the treatment outcomes of thyroid cancer have improved with advances in medical technology, early detection and effective treatment of the disease remain challenging, especially in resource-limited countries and regions. Moreover, environmental factors such radiation exposure and lifestyle changes such as obesity and dietary habits may also play a role in the rising incidence of thyroid cancer [8,23,25].

Our analysis of age distribution shows an increasing burden across all age groups, which was more pronounced in older populations. Older adults are more likely to have been exposed to a variety of thyroid cancer risk factors over their lifetime, including radiation, chronic diseases, and malnutrition [26], thereby contributing to the observed higher incidence in this population. The incidence of thyroid cancer increases with age, peaking at 79 years before showing variability in those aged 80–94-year-old. The trend in the DALY rate showed that the burden of life expectancy loss was higher in older age groups but tended to decrease in the ≥80-year-old age groups, possibly due to the prioritisation of other health problems and the diversity of causes of death in the elderly population [27]. These findings underscore the nuanced relationship between age, health status, and thyroid cancer risk [27,28].

While future trend projections suggest a decline in incidence and DALY rates for thyroid cancer across most age groups, a notable increase is anticipated among individuals >80 years old. This underscores the need for tailored public health interventions targeting older populations, emphasising early detection and management to mitigate the impact on health care systems and improve life expectancy [29,30]. Despite a generally low burden in the younger demographic, rising incidence rates highlight a significant risk of thyroid cancer among them, attributed to factors like genetic predispositions, unhealthy lifestyle choices, environmental pollution, and psychological stress [31,32]. The increase in diagnoses among young people is likely due to advancements in diagnostic technologies and heightened awareness of their health, underscoring the importance of early detection and prevention across all age groups [33].

We observed notable sex differences in the global burden of thyroid cancer between 1990 and 2019, with women consistently showing a higher disease burden than men, though the gap has narrowed over time [34–36]. Recent trends indicate an accelerated increase in the burden among men, possibly due to later diagnosis [37]. These findings highlight the need for gender-specific approaches in thyroid cancer prevention and treatment, emphasising risk factor awareness and the importance of regular screening for early detection [38].

Our finding of significant differences in thyroid cancer burden across regions underscores the necessity for region-specific public health strategies. For instance, in regions with high burdens, enhanced screening programs and public health campaigns focusing on lifestyle modifications could be instrumental in reducing incidence rates and improving the overall quality of health care; for example, Western Europe, Australasia, and Southern Latin America had relatively high burdens of thyroid cancer, whereas Southeast Asia, Central Latin America, and Eastern Sub-Saharan Africa had lower burdens. Specifically, in Western Europe, the high burden of thyroid cancer may be related to the region’s highly developed health care systems and the widespread availability of screening services for thyroid cancer [39]. Environmental factors in Western Europe, such as high levels of industrialisation and environmental pollution, may also have contributed to the increased burden of thyroid cancer. In Australasia, lifestyle may be an important factor influencing the burden of thyroid cancer. For example, sun exposure habits may increase the risk of certain types of cancer, although the direct correlation between sun exposure and thyroid cancer may require further research; in addition, the generally higher standard of living and health awareness in this region may have led to more cases being diagnosed [39,40].

In contrast, Southeast Asia experiences a relatively low reported burden of thyroid cancer, potentially masking undiagnosed or delayed cases due to limited medical resources. This underdiagnosis can stem from inadequate health awareness and screening facilities, contributing to an underestimation of thyroid cancer prevalence. Similarly, in regions like Central Latin America and Sub-Saharan Africa, factors like lower living standards, insufficient health care infrastructure, and limited thyroid cancer awareness could lead to early symptoms being ignored, resulting in undiagnosed cases that skew disease statistics. Economic challenges and public health system limitations further hinder these populations' ability to address and manage thyroid cancer risk.

In high-SDI regions, the ASIR trended downward; in contrast, both the ASIR and DALY rates trended upward in low-SDI and low-middle-SDI regions. In high-SDI regions, the burden of thyroid cancer may remain stable or increase slightly during 2020–30. These regions typically have better health care resources and higher levels of health awareness than other SDI regions, so thyroid cancer diagnosis and treatment are relatively timely and effective [41,42]. The observed disparities based on the SDI highlight the critical role of SDI in guiding public health strategies. Tailoring approaches to fit the specific needs and resources of each SDI category can greatly enhance the effectiveness of health care interventions in reducing the thyroid cancer burden.

While SDI serves as a valuable proxy for assessing health disparities and outcomes, we recognise that it may not fully explain the intricacies of thyroid cancer burden across different categories. This suggests the influence of additional factors beyond SDI. This understanding prompts a more comprehensive investigation into the complex determinants of thyroid cancer, advocating for a holistic approach to public health planning and intervention to address the multifaceted nature of its epidemiology seamlessly.

We predicted the relationship between thyroid cancer burden and SDI during 2020–30 using GAMs and analyzed its correlation with data from 1990 to 2019.Although the GBD database provides comprehensive data, the accuracy and completeness of the data may be limited by the reporting systems and data collection methods of each country. Additionally, the GAMs, although powerful, may be limited in their predictive accuracy by model assumptions, variability in historical data, and new trends or confounding factors that may emerge in the future. Although we explored the differences between different SDI regions, specific social, cultural, and economic factors within regions, which may have a significant impact on the thyroid cancer burden, may have been insufficiently analysed. Lastly, the differences in diagnostic efficiency among different countries cannot be adjusted for in any analysis based on GBD data.

## CONCLUSIONS

By analysing data from the past 30 years, we determined the global burden of thyroid cancer and predicted its trends over the next decade [43]. We found that the burden of thyroid cancer is on the rise globally, especially among women aged ≥80 years and in low- and medium-SDI regions, suggesting a need for targeted, effective prevention, diagnosis, and treatment of thyroid cancer, as well as for personalised medical and public health strategies for these populations and regions [44,45].

## Additional material


Online Supplementary Document
