# Supplementary material for: Trends and projections of the global burden of thyroid cancer from 1990 to 2030
Source: J Glob Health. 2024 May 17;14:04084. doi: 10.7189/jogh.14.04084 (PMC11109522; doi:10.7189/jogh.14.04084)
Supplement: Online Supplementary Document [file jogh-14-04084-s001.pdf]

Table S1. The numbers and age-standardised rate of thyroid cancer cases, deaths, and DALYs between 1990 and 2019, stratified by genders

Year and gender	ASIR	ASDR	Age-standardised DALYs
1990			
Both	87 582.64 (1.64)	22 966.38 (0.43)	667 462.46 (12.48)
Female	63 795.87 (2.4)	15 370.72 (0.58)	43 8427.43 (16.51)
Male	23 786.77 (0.88)	7595.65 (0.28)	229 035.03 (8.5)
1991			
Both	90 868.51 (1.67)	23 427.07 (0.43)	680 478.88 (12.53)
Female	66 092.31 (2.45)	15 629.41 (0.58)	445 622.3 (16.53)
Male	24 776.2 (0.91)	7797.66 (0.28)	234 856.58 (8.58)
1992			
Both	94 349.6 (1.71)	24 000.31 (0.44)	696 734.81 (12.63)
Female	68 547.39 (2.51)	15 997.25 (0.58)	455 902.17 (16.67)
Male	25 802.22 (0.93)	8003.07 (0.29)	240 832.64 (8.67)
1993			
Both	98 974.78 (1.77)	24 656.66 (0.44)	715 013.07 (12.78)
Female	71 888.33 (2.59)	16 398.46 (0.59)	466 718.22 (16.82)
Male	27 086.45 (0.96)	8258.19 (0.29)	248 294.85 (8.8)
1994			
Both	103 016.82 (1.82)	25 239.44 (0.44)	733 887.3 (12.93)
Female	74 751.42 (2.66)	16 722.83 (0.59)	477 874.68 (16.98)
Male	28 265.4 (0.99)	8516.61 (0.3)	256 012.62 (8.95)

1995			
Both	106 814.68 (1.86)	25 739.26 (0.45)	74 8649.1 (13.01)
Female	77 341.2 (2.71)	16 977.84 (0.6)	485 955.65 (17.03)
Male	29 473.48 (1.02)	8761.42 (0.3)	262 693.46 (9.05)
1996			
Both	110 373.89 (1.89)	26 205.49 (0.45)	761 624.31 (13.05)
Female	79 901.58 (2.76)	17 223.4 (0.6)	493 171.93 (17.05)
Male	30 472.31 (1.04)	8982.09 (0.31)	268 452.39 (9.12)
1997			
Both	114 216.38 (1.93)	26 754.02 (0.45)	777 475.88 (13.14)
Female	82 626.0 (2.82)	17 532.88 (0.6)	502 591.59 (17.13)
Male	31 590.38 (1.06)	9221.13 (0.31)	274 884.29 (9.21)
1998			
Both	118 717.95 (1.98)	27 348.04 (0.46)	794 553.88 (13.25)
Female	85 843.06 (2.89)	17 866.82 (0.6)	512 747.54 (17.24)
Male	32 874.89 (1.09)	9481.22 (0.31)	281 806.34 (9.32)
1999			
Both	123 615.89 (2.03)	28 006.03 (0.46)	813 865.89 (13.39)
Female	89 112.93 (2.96)	18 233.48 (0.61)	523 481.91 (17.37)
Male	34 502.96 (1.13)	9772.55 (0.32)	290 383.98 (9.48)
2000			
Both	128 373.57 (2.09)	28 649.81 (0.47)	832 310.05 (13.52)
Female	92 283.95 (3.02)	18 544.48 (0.61)	532 594.67 (17.44)
Male	36 089.62 (1.16)	10 105.33 (0.33)	299 715.38 (9.66)
2001			
Both	132 907.34 (2.13)	29 260.05 (0.47)	846 573.9 (13.58)

Female	95 199.09 (3.08)	18 849.19 (0.61)	539 075.06 (17.42)
Male	37 708.25 (1.2)	10 410.86 (0.33)	307 498.84 (9.79)
2002			
Both	138 024.21 (2.19)	30 034.74 (0.48)	863 435.93 (13.67)
Female	98 538.17 (3.14)	19 261.71 (0.61)	546 663.82 (17.44)
Male	39 486.04 (1.24)	10 773.03 (0.34)	316 772.11 (9.96)
2003			
Both	143 845.46 (2.25)	30 752.82 (0.48)	879 889.29 (13.75)
Female	102 388.16 (3.22)	19 600.23 (0.62)	553 327.99 (17.42)
Male	41 457.3 (1.29)	11 152.59 (0.35)	326 561.3 (10.14)
2004			
Both	149 202.93 (2.3)	31 340.76 (0.48)	894 919.87 (13.81)
Female	106 025.44 (3.29)	19 853.48 (0.62)	559 400.51 (17.38)
Male	43 177.5 (1.32)	11 487.28 (0.35)	335 519.36 (10.29)
2005			
Both	156 587.39 (2.39)	32 335.24 (0.49)	921 032.51 (14.04)
Female	111 089.18 (3.41)	20 389.76 (0.63)	573 220.27 (17.58)
Male	454 98.21 (1.38)	11 945.48 (0.36)	347 812.24 (10.53)
2006			
Both	162 485.34 (2.44)	33 227.47 (0.5)	942 956.03 (14.19)
Female	114 833.34 (3.48)	20 842.21 (0.63)	583578.69 (17.67)
Male	47 652.01 (1.43)	12 385.26 (0.37)	359 377.34 (10.75)
2007			
Both	169 570.83 (2.52)	34 135.39 (0.51)	966 149.69 (14.35)
Female	119 341.76 (3.57)	21 246.16 (0.63)	593 321.97 (17.73)
Male	50 229.07 (1.48)	12 889.23 (0.38)	372 827.73 (11.01)

2008			
Both	177 860.6 (2.61)	35 272.35 (0.52)	995 649.27 (14.6)
Female	124 391.46 (3.67)	21 725.36 (0.64)	605 104.42 (17.85)
Male	53 469.14 (1.56)	13 546.99 (0.4)	390 544.84 (11.39)
2009			
Both	184 899.42 (2.68)	36 165.87 (0.52)	1 018 104.64 (14.75)
Female	128 318.32 (3.74)	22 044.01 (0.64)	612 957.09 (17.86)
Male	56 581.1 (1.63)	14 121.86 (0.41)	405 147.55 (11.67)
2010			
Both	191 196.69 (2.74)	37 268.46 (0.53)	1 045 884.73 (14.97)
Female	131 855.4 (3.79)	22 524.71 (0.65)	625 210.7 (17.99)
Male	59 341.29 (1.69)	14 743.75 (0.42)	420 674.04 (11.98)
2011			
Both	195 015.54 (2.76)	38 122.42 (0.54)	1 063 260.31 (15.03)
Female	133 838.47 (3.8)	22 906.48 (0.65)	632 106.07 (17.96)
Male	61 177.07 (1.72)	15 215.94 (0.43)	431 154.24 (12.13)
2012			
Both	197 785.88 (2.76)	38 953.17 (0.54)	107 8502.2 (15.07)
Female	134 795.97 (3.78)	23 245.86 (0.65)	636 399.16 (17.87)
Male	62 989.91 (1.75)	15 707.32 (0.44)	442 103.04 (12.3)
2013			
Both	201 491.96 (2.78)	39 786.67 (0.55)	1 095 933.08 (15.13)
Female	136 950.28 (3.8)	23 676.11 (0.66)	645 701.08 (17.91)
Male	64 541.68 (1.77)	16 110.56 (0.44)	450 232.01 (12.38)
2014			
Both	204 779.3 (2.8)	40 408.7 (0.55)	1 107 138.48 (15.11)

Female	138 966.19 (3.81)	24 011.83 (0.66)	651 722.33 (17.86)
Male	65 813.12 (1.79)	16 396.87 (0.45)	455 416.15 (12.38)
2015			
Both	209 736.52 (2.83)	41 382.62 (0.56)	1 130 453.94 (15.26)
Female	142 090.88 (3.85)	24 585.09 (0.67)	665 415.53 (18.03)
Male	67 645.64 (1.82)	16 797.53 (0.45)	465 038.41 (12.5)
2016			
Both	214 507.6 (2.86)	42 347.45 (0.57)	1 153 595.25 (15.39)
Female	145 251.61 (3.89)	25 127.04 (0.67)	678 435.9 (18.17)
Male	69 255.99 (1.84)	17 220.41 (0.46)	475 159.35 (12.63)
2017			
Both	219 579.31 (2.9)	43 210.23 (0.57)	1 174 492.68 (15.5)
Female	148 463.58 (3.93)	25 612.23 (0.68)	690 085.56 (18.28)
Male	71 115.73 (1.87)	17 598.0 (0.46)	484 407.12 (12.74)
2018			
Both	227 155.35 (2.97)	44 374.57 (0.58)	1 203 523.46 (15.71)
Female	153 417.65 (4.02)	26 267.52 (0.69)	706 290.08 (18.51)
Male	73 737.7 (1.92)	18 107.05 (0.47)	497 233.38 (12.94)
2019			
Both	233 846.64 (3.02)	45 575.96 (0.59)	1 231 841.05 (15.92)
Female	157 832.91 (4.09)	26 940.76 (0.7)	721 999.18 (18.72)
Male	76 013.73 (1.96)	18 635.2 (0.48)	509 841.86 (13.14)

ASDR − age-standardised death rate, ASIR − age-standardised incidence rate, DALY − disability-adjusted life years

Table S2. The numbers and rates of thyroid cancer cases, deaths, and disability-adjusted life years between 1990 and 2030

	From 1990 to 2019						From 2020 to 2030					
	**ASIR**		**ASDR**		**Age-standardised DALY rate**		**ASIR**		**ASDR**		**Age-standardised DALY rate**	
**Location**	**EAPC (95% CI)**	***P*-value***	**EAPC (95% CI)**	***P*-value***	**EAPC (95% CI)**	***P*-value***	**EAPC (95% CI)**	***P*-value***	**EAPC (95% CI)**	***P*-value***	**EAPC (95% CI)**	***P*-value***
Afghanistan	0.34 (0.20, 0.48)	<0.001	−0.32 (−0.38, −0.25)	<0.001	−0.76 (−0.93, −0.58)	<0.001	1.08 (1.07, 1.09)	<0.001	0.01 (0.01, 0.01)	<0.001	0.35 (0.35, 0.35)	<0.001
Albania	2.28 (2.17, 2.38)	<0.001	0.65 (0.56, 0.74)	<0.001	1.21 (0.98, 1.44)	<0.001	1.73 (1.69, 1.76)	<0.001	0.97 (0.96, 0.98)	<0.001	1.54 (1.51, 1.56)	<0.001
Algeria	2.80 (2.72, 2.87)	<0.001	0.50 (0.47, 0.53)	<0.001	1.68 (1.58, 1.78)	<0.001	2.47 (2.40, 2.53)	<0.001	0.48 (0.48, 0.48)	<0.001	1.23 (1.22, 1.25)	<0.001
American Samoa	2.42 (2.21, 2.62)	<0.001	1.19 (1.10, 1.28)	<0.001	2.67 (2.45, 2.90)	<0.001	1.13 (1.11, 1.14)	<0.001	1.16 (1.15, 1.18)	<0.001	1.89 (1.85, 1.93)	<0.001
Andorra	1.91 (1.67, 2.14)	<0.001	0.66 (0.60, 0.72)	<0.001	0.98 (0.85, 1.11)	<0.001	1.17 (1.15, 1.18)	<0.001	0.55 (0.54, 0.55)	<0.001	1.40 (1.38, 1.42)	<0.001
Angola	0.24 (0.19, 0.29)	<0.001	−0.04 (−0.05, −0.02)	<0.001	−0.33 (−0.42, −0.25)	<0.001	0.86 (0.85, 0.87)	<0.001	0.20 (0.20, 0.20)	<0.001	0.89 (0.88, 0.90)	<0.001
Antigua and Barbuda	2.07 (1.76, 2.38)	<0.001	0.58 (0.36, 0.81)	<0.001	−0.33 (−0.42, −0.25)	<0.001	1.52 (1.49, 1.54)	<0.001	0.53 (0.53, 0.54)	<0.001	1.05 (1.04, 1.07)	<0.001
Argentina	0.27 (0.08, 0.46)	0.009	−0.27 (−0.37, −0.16)	<0.001	−0.83 (−1.13, −0.53)	<0.001	1.44 (1.42, 1.47)	<0.001	0.24 (0.24, 0.24)	<0.001	0.77 (0.76, 0.77)	<0.001
Armenia	3.39 (2.93, 3.84)	<0.001	1.62 (1.36, 1.88)	<0.001	3.99 (3.41, 4.56)	<0.001	−0.05 (−0.05, −0.05)	<0.001	−1.02 (−1.03, −1.01)	<0.001	−1.70 (−1.73, −1.67)	<0.001

Australia	2.79 (2.46, 3.11)	<0.001	0.81 (0.73, 0.89)	<0.001	1.82 (1.62, 2.03)	<0.001	−0.40 (−0.40, −0.40)	<0.001	0.28 (0.28, 0.28)	<0.001	0.10 (0.10, 0.10)	<0.001
Austria	−0.26 (−0.42, −0.09)	0.004	−0.61 (−0.69, −0.54)	<0.001	−1.33 (−1.47, −1.19)	<0.001	−0.09 (−0.09, −0.09)	<0.001	0.15 (0.15, 0.15)	<0.001	−0.08 (−0.08, −0.08)	<0.001
Azerbaijan	1.30 (1.13, 1.47)	<0.001	0.21 (0.17, 0.26)	<0.001	0.48 (0.29, 0.66)	<0.001	1.74 (1.71, 1.77)	<0.001	0.33 (0.33, 0.33)	<0.001	1.30 (1.29, 1.32)	<0.001
Bahamas	1.66 (1.58, 1.73)	<0.001	0.61 (0.53, 0.69)	<0.001	1.60 (1.42, 1.78)	<0.001	1.22 (1.21, 1.24)	<0.001	0.78 (0.78, 0.79)	<0.001	1.42 (1.40, 1.45)	<0.001
Bahrain	2.60 (2.40, 2.79)	<0.001	0.18 (0.10, 0.25)	<0.001	0.98 (0.73, 1.22)	<0.001	3.67 (3.53, 3.82)	<0.001	1.25 (1.24, 1.27)	<0.001	3.81 (3.65, 3.96)	<0.001
Bangladesh	1.46 (1.34, 1.59)	<0.001	0.37 (0.32, 0.41)	<0.001	0.91 (0.76, 1.05)	<0.001	2.51 (2.44, 2.58)	<0.001	0.97 (0.96, 0.98)	<0.001	1.97 (1.93, 2.01)	<0.001
Barbados	1.85 (1.76, 1.94)	<0.001	0.62 (0.55, 0.69)	<0.001	1.42 (1.29, 1.55)	<0.001	1.18 (1.16, 1.19)	<0.001	1.19 (1.17, 1.20)	<0.001	1.68 (1.65, 1.71)	<0.001
Belarus	2.23 (2.02, 2.45)	<0.001	0.76 (0.58, 0.94)	<0.001	1.14 (0.73, 1.55)	<0.001	2.06 (2.02, 2.11)	<0.001	0.43 (0.43, 0.43)	<0.001	1.34 (1.32, 1.35)	<0.001
Belgium	0.17 (−0.05, 0.39)	0.139	−0.88 (−1.19, −0.57)	<0.001	−1.36 (−1.77, −0.94)	<0.001	0.16 (0.16, 0.16)	<0.001	0.10 (0.10, 0.10)	<0.001	−0.04 (−0.04, −0.04)	<0.001
Belize	1.36 (1.31, 1.41)	<0.001	0.29 (0.26, 0.32)	<0.001	1.91 (1.77, 2.06)	<0.001	1.13 (1.12, 1.15)	<0.001	0.37 (0.37, 0.37)	<0.001	1.38 (1.36, 1.40)	<0.001
Benin	0.07 (0.05, 0.08)	<0.001	−0.06 (−0.06, −0.05)	<0.001	−0.31 (−0.36, −0.26)	<0.001	0.23 (0.23, 0.23)	<0.001	−0.04 (−0.04, −0.04)	<0.001	−0.28 (−0.28, −0.28)	<0.001
Bermuda	1.69 (1.56, 1.82)	<0.001	0.48 (0.28, 0.68)	<0.001	0.48 (0.15, 0.80)	0.008	0.74 (0.73, 0.74)	<0.001	0.97 (0.96, 0.98)	<0.001	0.98 (0.97, 0.99)	<0.001
Bhutan	1.92 (1.86, 1.99)	<0.001	0.54 (0.52, 0.57)	<0.001	1.14 (1.05, 1.23)	<0.001	2.42 (2.36, 2.49)	<0.001	0.93 (0.92, 0.94)	<0.001	1.88 (1.85, 1.92)	<0.001
Bolivia	1.93 (1.87, 1.99)	<0.001	0.89 (0.87, 0.91)	<0.001	1.24 (1.22, 1.26)	<0.001	2.13 (2.08, 2.18)	<0.001	0.74 (0.74, 0.75)	<0.001	1.14 (1.12, 1.15)	<0.001

Bosnia and Herzegovina	1.88 (1.67, 2.10)	<0.001	0.40 (0.33, 0.48)	<0.001	0.45 (0.26, 0.64)	<0.001	1.23 (1.22, 1.25)	<0.001	0.67 (0.67, 0.68)	<0.001	1.14 (1.13, 1.15)	<0.001
Botswana	0.41 (0.37, 0.46)	<0.001	0.05 (0.04, 0.07)	<0.001	0.72 (0.53, 0.92)	<0.001	0.61 (0.60, 0.61)	<0.001	0.11 (0.11, 0.11)	<0.001	1.00 (0.99, 1.01)	<0.001
Brazil	1.35 (1.17, 1.52)	<0.001	0.41 (0.38, 0.43)	<0.001	0.78 (0.66, 0.90)	<0.001	0.59 (0.59, 0.59)	<0.001	0.34 (0.34, 0.34)	<0.001	0.35 (0.35, 0.35)	<0.001
Brunei Darussalam	2.42 (2.23, 2.60)	<0.001	0.67 (0.57, 0.76)	<0.001	1.90 (1.62, 2.17)	<0.001	2.23 (2.18, 2.29)	<0.001	1.03 (1.02, 1.04)	<0.001	2.14 (2.09, 2.18)	<0.001
Bulgaria	0.80 (0.68, 0.93)	<0.001	0.14 (0.10, 0.17)	<0.001	−0.03 (−0.12, 0.06)	0.494	1.60 (1.57, 1.63)	<0.001	0.76 (0.75, 0.77)	<0.001	1.16 (1.15, 1.18)	<0.001
Burkina Faso	0.06 (0.03, 0.10)	0.002	−0.09 (−0.11, −0.06)	<0.001	−0.47 (−0.67, −0.27)	<0.001	0.40 (0.40, 0.41)	<0.001	0.05 (0.05, 0.05)	<0.001	0.55 (0.55, 0.56)	<0.001
Burundi	0.20 (0.15, 0.24)	<0.001	−0.20 (−0.24, −0.16)	<0.001	−0.58 (−0.70, −0.46)	<0.001	0.47 (0.47, 0.47)	<0.001	0.11 (0.11, 0.11)	<0.001	0.45 (0.44, 0.45)	<0.001
Cabo Verde	1.17 (1.11, 1.23)	<0.001	0.10 (0.05, 0.15)	<0.001	1.11 (0.93, 1.28)	<0.001	2.11 (2.06, 2.16)	<0.001	1.23 (1.21, 1.24)	<0.001	3.73 (3.58, 3.87)	<0.001
Cambodia	2.10 (1.99, 2.21)	<0.001	0.73 (0.67, 0.80)	<0.001	1.28 (1.17, 1.40)	<0.001	2.53 (2.46, 2.59)	<0.001	1.06 (1.05, 1.08)	<0.001	1.72 (1.69, 1.76)	<0.001
Cameroon	0.16 (0.14, 0.18)	<0.001	−0.02 (−0.03, −0.02)	<0.001	−0.06 (−0.11, −0.01)	0.021	0.48 (0.48, 0.49)	<0.001	−0.00 (−0.00, −0.00)	<0.001	−0.01 (−0.01, −0.01)	<0.001
Canada	1.27 (0.90, 1.64)	<0.001	0.54 (0.45, 0.64)	<0.001	0.90 (0.62, 1.17)	<0.001	0.33 (0.33, 0.33)	<0.001	0.80 (0.80, 0.81)	<0.001	1.00 (0.99, 1.01)	<0.001
Central African Republic	−0.05 (−0.08, −0.02)	0.009	−0.08 (−0.10, −0.06)	<0.001	−0.35 (−0.46, −0.24)	<0.001	−0.08 (−0.08, −0.08)	<0.001	−0.07 (−0.07, −0.07)	<0.001	−0.42 (−0.42, −0.42)	<0.001
Chad	−0.08 (−0.10, −0.07)	<0.001	−0.12 (−0.12, −0.11)	<0.001	−0.66 (−0.71, −0.61)	<0.001	0.07 (0.07, 0.07)	<0.001	−0.08 (−0.08, −0.08)	<0.001	−0.37 (−0.38, −0.37)	<0.001

Chile	2.40 (2.32, 2.48)	<0.001	0.66 (0.61, 0.72)	<0.001	1.15 (1.05, 1.26)	<0.001	0.91 (0.90, 0.92)	<0.001	0.76 (0.75, 0.76)	<0.001	1.01 (1.00, 1.02)	<0.001
China	2.62 (2.51, 2.74)	<0.001	0.66 (0.60, 0.72)	<0.001	1.49 (1.37, 1.62)	<0.001	2.13 (2.08, 2.18)	<0.001	0.55 (0.55, 0.55)	<0.001	1.13 (1.12, 1.14)	<0.001
Colombia	1.80 (1.60, 2.01)	<0.001	0.57 (0.42, 0.71)	<0.001	0.71 (0.36, 1.06)	<0.001	1.32 (1.30, 1.34)	<0.001	0.72 (0.72, 0.73)	<0.001	0.89 (0.88, 0.89)	<0.001
Comoros	1.42 (1.25, 1.59)	<0.001	0.55 (0.49, 0.61)	<0.001	1.52 (1.23, 1.80)	<0.001	−0.06 (−0.06, −0.06)	<0.001	0.08 (0.08, 0.08)	<0.001	−1.07 (−1.08, −1.06)	<0.001
Congo	0.29 (0.21, 0.37)	<0.001	−0.10 (−0.14, −0.06)	<0.001	−0.42 (−0.59, −0.25)	<0.001	0.86 (0.85, 0.86)	<0.001	0.25 (0.25, 0.25)	<0.001	0.97 (0.96, 0.98)	<0.001
Cook Islands	0.58 (0.27, 0.89)	<0.001	0.11 (−0.07, 0.29)	0.233	−0.24 (−0.70, 0.22)	0.306	1.79 (1.75, 1.82)	<0.001	0.64 (0.63, 0.64)	<0.001	1.23 (1.22, 1.25)	<0.001
Costa Rica	1.51 (1.30, 1.73)	<0.001	0.53 (0.42, 0.63)	<0.001	1.00 (0.74, 1.27)	<0.001	1.97 (1.93, 2.01)	<0.001	1.04 (1.02, 1.05)	<0.001	1.91 (1.87, 1.95)	<0.001
Croatia	0.63 (0.36, 0.90)	<0.001	−0.00 (−0.15, 0.15)	0.995	−0.54 (−0.82, −0.26)	<0.001	0.16 (0.16, 0.16)	<0.001	0.21 (0.21, 0.21)	<0.001	0.03 (0.03, 0.03)	<0.001
Cuba	2.38 (2.24, 2.52)	<0.001	1.14 (1.01, 1.26)	<0.001	2.28 (2.06, 2.51)	<0.001	1.21 (1.19, 1.22)	<0.001	0.51 (0.51, 0.51)	<0.001	0.84 (0.83, 0.85)	<0.001
Cyprus	0.94 (0.66, 1.22)	<0.001	−0.43 (−0.48, −0.38)	<0.001	−1.06 (−1.21, −0.91)	<0.001	0.95 (0.94, 0.96)	<0.001	0.62 (0.61, 0.62)	<0.001	1.07 (1.06, 1.08)	<0.001
Czechia	0.21 (−0.03, 0.45)	0.095	−0.42 (−0.51, −0.34)	<0.001	−1.08 (−1.21, −0.94)	<0.001	0.93 (0.92, 0.94)	<0.001	0.65 (0.65, 0.66)	<0.001	0.94 (0.93, 0.95)	<0.001
Côte d'Ivoire	0.10 (0.09, 0.11)	<0.001	−0.00 (−0.01, 0.01)	0.973	−0.15 (−0.25, −0.04)	0.009	0.35 (0.34, 0.35)	<0.001	0.03 (0.03, 0.03)	<0.001	0.04 (0.04, 0.04)	<0.001

Democratic People's Republic of Korea	0.61 (0.42, 0.79)	<0.001	0.45 (0.43, 0.46)	<0.001	0.66 (0.63, 0.69)	<0.001	1.10 (1.08, 1.11)	<0.001	0.35 (0.35, 0.35)	<0.001	0.55 (0.55, 0.56)	<0.001
Democratic Republic of the Congo	0.04 (−0.00, 0.08)	0.073	−0.06 (−0.07, −0.05)	<0.001	−0.40 (−0.49, −0.30)	<0.001	0.67 (0.66, 0.67)	<0.001	0.13 (0.13, 0.13)	<0.001	0.67 (0.66, 0.67)	<0.001
Denmark	1.85 (1.56, 2.14)	<0.001	0.54 (0.42, 0.66)	<0.001	0.99 (0.68, 1.30)	<0.001	0.36 (0.36, 0.36)	<0.001	0.24 (0.24, 0.24)	<0.001	0.27 (0.27, 0.28)	<0.001
Djibouti	1.54 (1.42, 1.65)	<0.001	0.54 (0.52, 0.56)	<0.001	2.05 (1.94, 2.16)	<0.001	2.40 (2.34, 2.46)	<0.001	0.82 (0.81, 0.83)	<0.001	2.27 (2.22, 2.33)	<0.001
Dominica	1.17 (1.12, 1.23)	<0.001	0.60 (0.53, 0.67)	<0.001	1.44 (1.27, 1.60)	<0.001	0.54 (0.54, 0.54)	<0.001	0.38 (0.38, 0.38)	<0.001	0.88 (0.87, 0.89)	<0.001
Dominican Republic	2.29 (2.15, 2.42)	<0.001	0.94 (0.84, 1.04)	<0.001	2.81 (2.50, 3.12)	<0.001	1.65 (1.62, 1.68)	<0.001	0.45 (0.44, 0.45)	<0.001	0.90 (0.89, 0.90)	<0.001
Ecuador	3.60 (3.23, 3.98)	<0.001	1.84 (1.68, 2.00)	<0.001	3.32 (2.91, 3.73)	<0.001	1.23 (1.21, 1.25)	<0.001	0.03 (0.03, 0.03)	<0.001	0.28 (0.28, 0.28)	<0.001
Egypt	1.29 (1.13, 1.46)	<0.001	0.13 (0.11, 0.15)	<0.001	0.28 (0.07, 0.50)	0.016	2.91 (2.82, 3.00)	<0.001	0.15 (0.15, 0.15)	<0.001	1.09 (1.08, 1.10)	<0.001
El Salvador	0.67 (0.36, 0.99)	<0.001	−0.19 (−0.41, 0.04)	0.11	−0.83 (−1.48, −0.18)	0.018	1.72 (1.69, 1.75)	<0.001	0.72 (0.71, 0.72)	<0.001	1.46 (1.44, 1.49)	<0.001
Equatorial Guinea	0.37 (0.29, 0.46)	<0.001	−0.20 (−0.25, −0.14)	<0.001	−1.18 (−1.49, −0.87)	<0.001	0.92 (0.91, 0.93)	<0.001	0.17 (0.17, 0.17)	<0.001	0.95 (0.94, 0.95)	<0.001
Eritrea	1.19 (1.13, 1.25)	<0.001	0.45 (0.43, 0.47)	<0.001	1.55 (1.46, 1.63)	<0.001	1.50 (1.48, 1.53)	<0.001	0.44 (0.44, 0.45)	<0.001	1.35 (1.33, 1.37)	<0.001
Estonia	1.61 (1.43, 1.78)	<0.001	0.61 (0.50, 0.72)	<0.001	0.08 (−0.16, 0.33)	0.501	0.14 (0.14, 0.14)	<0.001	0.24 (0.24, 0.24)	<0.001	0.07 (0.07, 0.07)	<0.001
Eswatini	0.76 (0.65, 0.87)	<0.001	0.35 (0.25, 0.45)	<0.001	1.59 (1.09, 2.09)	<0.001	0.96 (0.95, 0.97)	<0.001	0.33 (0.32, 0.33)	<0.001	0.88 (0.87, 0.89)	<0.001

Ethiopia	−0.50 (−0.72, −0.28)	<0.001	−0.95 (−1.08, −0.83)	<0.001	−2.33 (−2.55, −2.10)	<0.001	2.21 (2.16, 2.26)	<0.001	0.49 (0.49, 0.49)	<0.001	0.97 (0.96, 0.98)	<0.001
Fiji	1.12 (1.00, 1.24)	<0.001	0.57 (0.47, 0.68)	<0.001	0.87 (0.72, 1.03)	<0.001	2.25 (2.20, 2.30)	<0.001	1.22 (1.21, 1.24)	<0.001	1.82 (1.78, 1.85)	<0.001
Finland	1.69 (1.42, 1.97)	<0.001	0.49 (0.41, 0.58)	<0.001	0.57 (0.40, 0.74)	<0.001	0.26 (0.25, 0.26)	<0.001	0.42 (0.42, 0.43)	<0.001	0.19 (0.19, 0.19)	<0.001
France	0.50 (−0.11, 1.10)	0.119	−0.41 (−0.53, −0.29)	<0.001	−0.93 (−1.40, −0.47)	<0.001	1.08 (1.07, 1.09)	<0.001	1.03 (1.02, 1.04)	<0.001	1.51 (1.48, 1.53)	<0.001
Gabon	0.35 (0.29, 0.40)	<0.001	−0.07 (−0.09, −0.05)	<0.001	−0.22 (−0.30, −0.14)	<0.001	1.39 (1.37, 1.42)	<0.001	0.34 (0.34, 0.34)	<0.001	1.24 (1.23, 1.26)	<0.001
Gambia	0.35 (0.30, 0.41)	<0.001	0.12 (0.10, 0.15)	<0.001	0.83 (0.64, 1.02)	<0.001	0.83 (0.83, 0.84)	<0.001	0.18 (0.18, 0.18)	<0.001	1.31 (1.29, 1.33)	<0.001
Georgia	2.37 (2.15, 2.60)	<0.001	1.32 (1.20, 1.45)	<0.001	3.05 (2.73, 3.36)	<0.001	0.65 (0.64, 0.65)	<0.001	−0.54 (−0.55, −0.54)	<0.001	−0.79 (−0.80, −0.79)	<0.001
Germany	0.49 (0.35, 0.63)	<0.001	−0.27 (−0.43, −0.12)	0.002	−0.72 (−0.95, −0.49)	<0.001	−0.30 (−0.30, −0.29)	<0.001	0.19 (0.19, 0.19)	<0.001	−0.22 (−0.22, −0.22)	<0.001
Ghana	0.19 (0.17, 0.21)	<0.001	0.06 (0.05, 0.07)	<0.001	0.40 (0.28, 0.53)	<0.001	0.45 (0.45, 0.46)	<0.001	0.05 (0.05, 0.05)	<0.001	0.07 (0.07, 0.07)	<0.001
Greece	0.80 (0.61, 0.99)	<0.001	0.08 (−0.00, 0.17)	0.076	−0.04 (−0.17, 0.10)	0.587	1.38 (1.36, 1.40)	<0.001	1.48 (1.45, 1.50)	<0.001	1.78 (1.75, 1.81)	<0.001
Greenland	1.53 (1.32, 1.75)	<0.001	0.87 (0.75, 0.99)	<0.001	1.67 (1.39, 1.96)	<0.001	0.72 (0.72, 0.73)	<0.001	0.46 (0.45, 0.46)	<0.001	0.63 (0.63, 0.64)	<0.001
Grenada	1.11 (0.83, 1.39)	<0.001	0.20 (−0.02, 0.42)	0.083	0.74 (0.33, 1.14)	0.001	0.16 (0.16, 0.16)	<0.001	−0.05 (−0.05, −0.05)	<0.001	−1.00 (−1.01, −0.99)	<0.001

Guam	3.13 (2.63, 3.63)	<0.001	1.49 (1.38, 1.59)	<0.001	4.45 (3.98, 4.93)	<0.001	1.32 (1.30, 1.33)	<0.001	1.32 (1.30, 1.33)	<0.001	1.73 (1.70, 1.77)	<0.001
Guatemala	0.58 (0.34, 0.81)	<0.001	−0.11 (−0.27, 0.04)	0.155	−0.65 (−1.14, −0.15)	0.016	1.19 (1.18, 1.20)	<0.001	0.28 (0.28, 0.29)	<0.001	0.65 (0.65, 0.66)	<0.001
Guinea	0.20 (0.17, 0.23)	<0.001	−0.04 (−0.08, −0.01)	0.014	0.06 (−0.02, 0.13)	0.138	0.16 (0.16, 0.16)	<0.001	−0.31 (−0.31, −0.31)	<0.001	−1.08 (−1.10, −1.07)	<0.001
Guinea-Bissau	0.01 (−0.01, 0.04)	0.4	−0.11 (−0.11, −0.10)	<0.001	−0.61 (−0.65, −0.56)	<0.001	0.18 (0.18, 0.18)	<0.001	−0.06 (−0.06, −0.06)	<0.001	−0.53 (−0.53, −0.52)	<0.001
Guyana	1.51 (1.43, 1.59)	<0.001	0.67 (0.62, 0.73)	<0.001	2.02 (1.90, 2.14)	<0.001	1.37 (1.35, 1.39)	<0.001	0.31 (0.31, 0.31)	<0.001	0.63 (0.63, 0.64)	<0.001
Haiti	0.43 (0.34, 0.52)	<0.001	−0.03 (−0.08, 0.03)	0.377	−0.09 (−0.29, 0.10)	0.341	0.69 (0.69, 0.70)	<0.001	0.19 (0.19, 0.19)	<0.001	0.39 (0.39, 0.39)	<0.001
Honduras	2.85 (2.71, 3.00)	<0.001	1.68 (1.59, 1.77)	<0.001	2.45 (2.30, 2.61)	<0.001	1.84 (1.80, 1.87)	<0.001	0.39 (0.39, 0.40)	<0.001	0.80 (0.79, 0.80)	<0.001
Hungary	0.22 (0.10, 0.34)	0.001	−0.43 (−0.59, −0.27)	<0.001	−1.10 (−1.34, −0.87)	<0.001	1.34 (1.32, 1.36)	<0.001	0.43 (0.43, 0.43)	<0.001	0.66 (0.66, 0.67)	<0.001
Iceland	0.69 (0.40, 0.99)	<0.001	0.07 (−0.02, 0.16)	0.146	−0.13 (−0.26, 0.00)	0.064	0.38 (0.38, 0.38)	<0.001	0.46 (0.46, 0.46)	<0.001	0.43 (0.43, 0.43)	<0.001
India	1.58 (1.53, 1.64)	<0.001	0.53 (0.51, 0.56)	<0.001	1.33 (1.21, 1.45)	<0.001	2.31 (2.26, 2.37)	<0.001	0.81 (0.80, 0.82)	<0.001	1.90 (1.86, 1.93)	<0.001
Indonesia	1.73 (1.68, 1.78)	<0.001	0.72 (0.70, 0.74)	<0.001	1.42 (1.34, 1.50)	<0.001	1.93 (1.89, 1.97)	<0.001	0.78 (0.77, 0.78)	<0.001	1.45 (1.43, 1.47)	<0.001
Iran	3.19 (3.01, 3.37)	<0.001	0.69 (0.60, 0.78)	<0.001	2.85 (2.54, 3.16)	<0.001	2.62 (2.54, 2.69)	<0.001	0.47 (0.47, 0.47)	<0.001	1.46 (1.44, 1.48)	<0.001
Iraq	2.67 (2.36, 2.97)	<0.001	0.43 (0.34, 0.52)	<0.001	1.75 (1.46, 2.05)	<0.001	1.65 (1.62, 1.68)	<0.001	0.31 (0.31, 0.32)	<0.001	0.35 (0.35, 0.35)	<0.001

Ireland	1.91 (1.74, 2.09)	<0.001	−0.06 (−0.19, 0.08)	0.417	0.01 (−0.25, 0.27)	0.952	0.10 (0.10, 0.10)	<0.001	0.34 (0.34, 0.34)	<0.001	0.35 (0.35, 0.35)	<0.001
Israel	1.65 (1.33, 1.98)	<0.001	0.15 (0.04, 0.25)	0.009	0.15 (−0.09, 0.38)	0.238	0.72 (0.71, 0.72)	<0.001	0.37 (0.37, 0.37)	<0.001	0.60 (0.60, 0.61)	<0.001
Italy	0.28 (0.05, 0.51)	0.024	−0.05 (−0.09, −0.02)	0.008	−0.62 (−0.70, −0.54)	<0.001	0.90 (0.89, 0.90)	<0.001	0.81 (0.80, 0.82)	<0.001	1.09 (1.08, 1.10)	<0.001
Jamaica	2.69 (2.43, 2.96)	<0.001	0.98 (0.89, 1.07)	<0.001	2.93 (2.64, 3.23)	<0.001	1.13 (1.12, 1.15)	<0.001	0.74 (0.73, 0.74)	<0.001	1.41 (1.39, 1.43)	<0.001
Japan	1.70 (1.48, 1.92)	<0.001	1.33 (1.30, 1.36)	<0.001	1.16 (1.03, 1.29)	<0.001	−0.08 (−0.08, −0.08)	<0.001	1.15 (1.14, 1.16)	<0.001	0.73 (0.72, 0.73)	<0.001
Jordan	1.11 (0.89, 1.32)	<0.001	−0.08 (−0.14, −0.02)	0.017	−0.62 (−0.92, −0.32)	<0.001	2.49 (2.42, 2.56)	<0.001	0.73 (0.73, 0.74)	<0.001	2.52 (2.45, 2.58)	<0.001
Kazakhstan	−0.11 (−0.38, 0.15)	0.398	−0.80 (−0.95, −0.65)	<0.001	−2.17 (−2.53, −1.81)	<0.001	1.35 (1.33, 1.37)	<0.001	−0.01 (−0.01, −0.01)	<0.001	0.26 (0.26, 0.26)	<0.001
Kenya	0.70 (0.66, 0.74)	<0.001	0.24 (0.22, 0.27)	<0.001	1.98 (1.78, 2.17)	<0.001	1.01 (1.00, 1.02)	<0.001	0.15 (0.15, 0.15)	<0.001	0.73 (0.72, 0.73)	<0.001
Kiribati	0.14 (0.11, 0.17)	<0.001	−0.04 (−0.05, −0.03)	<0.001	−0.19 (−0.24, −0.13)	<0.001	0.63 (0.63, 0.64)	<0.001	0.14 (0.14, 0.14)	<0.001	0.67 (0.66, 0.67)	<0.001
Kuwait	0.72 (0.37, 1.07)	<0.001	0.09 (−0.01, 0.19)	0.086	−0.01 (−0.41, 0.38)	0.945	3.58 (3.45, 3.72)	<0.001	0.82 (0.82, 0.83)	<0.001	3.33 (3.21, 3.45)	<0.001
Kyrgyzstan	0.44 (0.26, 0.62)	<0.001	−0.19 (−0.23, −0.15)	<0.001	−0.65 (−0.84, −0.46)	<0.001	0.99 (0.98, 1.00)	<0.001	−0.00 (−0.00, −0.00)	<0.001	0.20 (0.20, 0.20)	<0.001

Lao People's Democratic Republic	0.80 (0.66, 0.94)	<0.001	−0.12 (−0.19, −0.06)	0.001	−0.50 (−0.66, −0.33)	<0.001	1.86 (1.83, 1.90)	<0.001	0.64 (0.63, 0.64)	<0.001	1.18 (1.16, 1.19)	<0.001
Latvia	1.88 (1.61, 2.15)	<0.001	1.07 (0.89, 1.25)	<0.001	1.18 (0.80, 1.56)	<0.001	1.08 (1.06, 1.09)	<0.001	0.15 (0.15, 0.15)	<0.001	0.09 (0.09, 0.09)	<0.001
Lebanon	3.52 (3.39, 3.65)	<0.001	0.54 (0.47, 0.61)	<0.001	1.31 (1.19, 1.44)	<0.001	2.26 (2.20, 2.31)	<0.001	0.47 (0.47, 0.48)	<0.001	1.33 (1.31, 1.34)	<0.001
Lesotho	1.11 (1.00, 1.21)	<0.001	0.58 (0.51, 0.65)	<0.001	2.62 (2.31, 2.93)	<0.001	0.41 (0.41, 0.41)	<0.001	−0.15 (−0.15, −0.15)	<0.001	−0.35 (−0.35, −0.35)	<0.001
Liberia	0.04 (−0.03, 0.11)	0.286	−0.18 (−0.23, −0.14)	<0.001	−0.85 (−1.17, −0.53)	<0.001	0.42 (0.42, 0.42)	<0.001	0.05 (0.05, 0.05)	<0.001	0.58 (0.57, 0.58)	<0.001
Libya	2.79 (2.48, 3.10)	<0.001	0.38 (0.31, 0.45)	<0.001	1.80 (1.52, 2.08)	<0.001	1.57 (1.54, 1.59)	<0.001	0.81 (0.81, 0.82)	<0.001	2.16 (2.11, 2.21)	<0.001
Lithuania	0.67 (0.25, 1.10)	0.004	0.41 (0.20, 0.61)	<0.001	0.09 (−0.39, 0.57)	0.719	0.55 (0.54, 0.55)	<0.001	0.01 (0.01, 0.01)	<0.001	−0.21 (−0.21, −0.21)	<0.001
Luxembourg	−0.00 (−0.25, 0.24)	0.976	−0.91 (−0.94, −0.88)	<0.001	−1.68 (−1.76, −1.60)	<0.001	0.11 (0.11, 0.11)	<0.001	0.10 (0.10, 0.10)	<0.001	0.24 (0.24, 0.24)	<0.001
Madagascar	0.45 (0.35, 0.54)	<0.001	0.10 (0.06, 0.14)	<0.001	0.38 (0.16, 0.60)	0.002	1.19 (1.17, 1.20)	<0.001	0.32 (0.32, 0.32)	<0.001	1.01 (1.00, 1.02)	<0.001
Malawi	0.37 (0.32, 0.42)	<0.001	−0.01 (−0.03, 0.01)	0.369	−0.16 (−0.26, −0.06)	0.004	1.11 (1.09, 1.12)	<0.001	0.17 (0.17, 0.17)	<0.001	0.74 (0.73, 0.74)	<0.001
Malaysia	2.15 (2.04, 2.26)	<0.001	0.49 (0.43, 0.54)	<0.001	1.01 (0.86, 1.16)	<0.001	3.15 (3.04, 3.25)	<0.001	1.30 (1.28, 1.32)	<0.001	2.58 (2.51, 2.65)	<0.001
Maldives	1.74 (1.56, 1.91)	<0.001	−0.09 (−0.12, −0.05)	<0.001	−0.73 (−0.93, −0.53)	<0.001	2.86 (2.77, 2.95)	<0.001	0.76 (0.75, 0.76)	<0.001	2.45 (2.39, 2.52)	<0.001

Mali	0.05 (0.02, 0.08)	0.004	−0.14 (−0.15, −0.12)	<0.001	−0.69 (−0.79, −0.59)	<0.001	0.50 (0.50, 0.50)	<0.001	−0.03 (−0.03, −0.03)	<0.001	0.23 (0.23, 0.23)	<0.001
Malta	1.52 (1.23, 1.81)	<0.001	0.33 (0.27, 0.39)	<0.001	0.39 (0.22, 0.55)	<0.001	1.26 (1.24, 1.28)	<0.001	0.94 (0.93, 0.95)	<0.001	1.06 (1.05, 1.08)	<0.001
Marshall Islands	1.66 (1.61, 1.70)	<0.001	0.58 (0.55, 0.60)	<0.001	2.07 (1.97, 2.17)	<0.001	1.62 (1.59, 1.65)	<0.001	0.83 (0.82, 0.83)	<0.001	1.61 (1.58, 1.64)	<0.001
Mauritania	0.05 (0.01, 0.10)	0.022	−0.13 (−0.15, −0.11)	<0.001	−0.89 (−1.02, −0.76)	<0.001	0.42 (0.42, 0.42)	<0.001	0.02 (0.02, 0.02)	<0.001	−0.21 (−0.21, −0.21)	<0.001
Mauritius	1.82 (1.62, 2.02)	<0.001	0.60 (0.51, 0.69)	<0.001	1.88 (1.56, 2.20)	<0.001	1.70 (1.67, 1.73)	<0.001	0.87 (0.86, 0.88)	<0.001	1.73 (1.70, 1.76)	<0.001
Mexico	2.32 (2.25, 2.39)	<0.001	0.86 (0.79, 0.93)	<0.001	2.12 (1.96, 2.27)	<0.001	2.23 (2.18, 2.29)	<0.001	1.08 (1.07, 1.09)	<0.001	2.06 (2.01, 2.10)	<0.001
Micronesia (Federated States of)	1.77 (1.71, 1.83)	<0.001	0.53 (0.49, 0.56)	<0.001	1.48 (1.43, 1.53)	<0.001	1.99 (1.95, 2.04)	<0.001	1.02 (1.01, 1.03)	<0.001	1.93 (1.89, 1.97)	<0.001
Monaco	1.14 (1.02, 1.25)	<0.001	0.08 (0.05, 0.11)	<0.001	0.17 (0.13, 0.21)	<0.001	−0.76 (−0.76, −0.75)	<0.001	−0.26 (−0.26, −0.26)	<0.001	−0.57 (−0.57, −0.56)	<0.001
Mongolia	1.54 (1.44, 1.64)	<0.001	0.37 (0.35, 0.40)	<0.001	1.60 (1.53, 1.68)	<0.001	1.90 (1.86, 1.94)	<0.001	0.69 (0.68, 0.69)	<0.001	2.05 (2.00, 2.09)	<0.001
Montenegro	1.74 (1.66, 1.81)	<0.001	0.70 (0.63, 0.77)	<0.001	1.07 (0.88, 1.25)	<0.001	0.97 (0.96, 0.98)	<0.001	0.18 (0.18, 0.18)	<0.001	0.49 (0.48, 0.49)	<0.001
Morocco	2.30 (2.25, 2.34)	<0.001	0.52 (0.49, 0.56)	<0.001	1.21 (1.15, 1.27)	<0.001	2.48 (2.42, 2.55)	<0.001	0.67 (0.66, 0.67)	<0.001	1.33 (1.31, 1.35)	<0.001
Mozambique	0.98 (0.87, 1.08)	<0.001	0.29 (0.23, 0.35)	<0.001	1.15 (0.91, 1.39)	<0.001	0.78 (0.78, 0.79)	<0.001	−0.03 (−0.03, −0.03)	<0.001	−0.05 (−0.05, −0.05)	<0.001
Myanmar	1.26 (1.18, 1.34)	<0.001	0.32 (0.27, 0.37)	<0.001	0.24 (0.12, 0.36)	<0.001	2.10 (2.05, 2.15)	<0.001	0.68 (0.68, 0.69)	<0.001	1.14 (1.13, 1.16)	<0.001
Namibia	1.08 (1.00, 1.17)	<0.001	0.21 (0.18, 0.23)	<0.001	1.01 (0.85, 1.16)	<0.001	1.18 (1.17, 1.19)	<0.001	0.24 (0.24, 0.24)	<0.001	0.79 (0.78, 0.79)	<0.001

Nauru	1.03 (0.91, 1.15)	<0.001	0.11 (0.04, 0.18)	0.004	0.66 (0.40, 0.93)	<0.001	2.02 (1.98, 2.06)	<0.001	0.21 (0.21, 0.21)	<0.001	0.55 (0.55, 0.55)	<0.001
Nepal	1.54 (1.39, 1.70)	<0.001	0.55 (0.46, 0.64)	<0.001	1.27 (0.97, 1.56)	<0.001	2.08 (2.03, 2.13)	<0.001	0.81 (0.80, 0.81)	<0.001	1.62 (1.59, 1.64)	<0.001
Netherlands	1.94 (1.82, 2.06)	<0.001	0.54 (0.45, 0.64)	<0.001	0.96 (0.81, 1.12)	<0.001	0.19 (0.19, 0.19)	<0.001	0.41 (0.41, 0.41)	<0.001	0.43 (0.42, 0.43)	<0.001
New Zealand	1.91 (1.79, 2.02)	<0.001	0.96 (0.90, 1.01)	<0.001	1.78 (1.63, 1.93)	<0.001	0.78 (0.78, 0.79)	<0.001	0.92 (0.91, 0.93)	<0.001	1.18 (1.17, 1.19)	<0.001
Nicaragua	2.59 (2.47, 2.71)	<0.001	0.88 (0.82, 0.95)	<0.001	2.96 (2.74, 3.19)	<0.001	1.48 (1.45, 1.50)	<0.001	0.61 (0.60, 0.61)	<0.001	1.27 (1.25, 1.28)	<0.001
Niger	0.04 (0.04, 0.05)	<0.001	−0.00 (−0.00, 0.00)	0.243	0.13 (0.07, 0.19)	<0.001	0.03 (0.03, 0.03)	<0.001	−0.00 (−0.00, −0.00)	<0.001	−0.15 (−0.15, −0.15)	<0.001
Nigeria	0.15 (0.12, 0.18)	<0.001	−0.05 (−0.05, −0.04)	<0.001	−0.21 (−0.28, −0.14)	<0.001	0.15 (0.15, 0.15)	<0.001	−0.02 (−0.02, −0.02)	<0.001	−0.28 (−0.28, −0.28)	<0.001
Niue	1.85 (1.61, 2.09)	<0.001	0.44 (0.30, 0.58)	<0.001	0.99 (0.69, 1.29)	<0.001	0.83 (0.82, 0.83)	<0.001	0.14 (0.14, 0.14)	<0.001	0.38 (0.38, 0.38)	<0.001
North Macedonia	1.05 (0.79, 1.30)	<0.001	−0.05 (−0.18, 0.08)	0.466	−0.46 (−0.81, −0.11)	0.015	1.20 (1.18, 1.21)	<0.001	0.48 (0.48, 0.48)	<0.001	1.01 (1.00, 1.02)	<0.001
Northern Mariana Islands	3.67 (3.44, 3.89)	<0.001	1.76 (1.52, 1.99)	<0.001	5.39 (4.89, 5.88)	<0.001	2.27 (2.21, 2.32)	<0.001	1.90 (1.86, 1.93)	<0.001	2.72 (2.64, 2.80)	<0.001
Norway	1.44 (1.28, 1.61)	<0.001	0.10 (0.06, 0.13)	<0.001	0.12 (0.03, 0.21)	0.016	−0.46 (−0.46, −0.46)	<0.001	−0.08 (−0.08, −0.08)	<0.001	−0.22 (−0.22, −0.22)	<0.001
Oman	2.38 (2.07, 2.70)	<0.001	0.14 (0.08, 0.20)	<0.001	1.18 (0.83, 1.52)	<0.001	0.95 (0.94, 0.96)	<0.001	−0.24 (−0.24, −0.24)	<0.001	−0.66 (−0.67, −0.66)	<0.001
Pakistan	1.58 (1.51, 1.65)	<0.001	0.28 (0.22, 0.35)	<0.001	1.12 (0.89, 1.35)	<0.001	1.59 (1.56, 1.61)	<0.001	0.19 (0.19, 0.19)	<0.001	0.40 (0.40, 0.40)	<0.001

Palau	2.37 (2.23, 2.50)	<0.001	0.98 (0.89, 1.06)	<0.001	1.96 (1.89, 2.03)	<0.001	2.42 (2.35, 2.48)	<0.001	1.66 (1.63, 1.69)	<0.001	2.35 (2.29, 2.40)	<0.001
Palestine	1.13 (0.74, 1.53)	<0.001	0.02 (−0.08, 0.11)	0.731	0.28 (−0.14, 0.69)	0.204	2.91 (2.82, 3.00)	<0.001	0.50 (0.50, 0.50)	<0.001	2.04 (2.00, 2.09)	<0.001
Panama	1.66 (1.55, 1.77)	<0.001	0.66 (0.60, 0.72)	<0.001	1.54 (1.33, 1.76)	<0.001	2.05 (2.00, 2.09)	<0.001	0.39 (0.39, 0.40)	<0.001	0.97 (0.96, 0.98)	<0.001
Papua New Guinea	0.59 (0.56, 0.62)	<0.001	0.14 (0.13, 0.15)	<0.001	0.72 (0.68, 0.77)	<0.001	0.96 (0.96, 0.97)	<0.001	0.28 (0.28, 0.28)	<0.001	1.02 (1.01, 1.03)	<0.001
Paraguay	2.08 (1.98, 2.18)	<0.001	0.80 (0.77, 0.84)	<0.001	2.21 (2.08, 2.34)	<0.001	2.37 (2.31, 2.43)	<0.001	0.60 (0.59, 0.60)	<0.001	1.58 (1.55, 1.61)	<0.001
Peru	2.35 (2.23, 2.47)	<0.001	0.67 (0.59, 0.74)	<0.001	1.15 (0.98, 1.32)	<0.001	1.73 (1.70, 1.76)	<0.001	0.51 (0.51, 0.52)	<0.001	0.96 (0.95, 0.97)	<0.001
Philippines	0.77 (0.57, 0.97)	<0.001	0.34 (0.22, 0.46)	<0.001	0.54 (0.30, 0.78)	<0.001	1.45 (1.42, 1.47)	<0.001	0.43 (0.43, 0.44)	<0.001	0.71 (0.71, 0.72)	<0.001
Poland	−0.15 (−0.38, 0.08)	0.203	−0.76 (−0.98, −0.54)	<0.001	−1.99 (−2.43, −1.55)	<0.001	1.00 (0.99, 1.01)	<0.001	0.71 (0.71, 0.72)	<0.001	1.01 (1.00, 1.02)	<0.001
Portugal	0.77 (0.55, 1.00)	<0.001	−0.36 (−0.59, −0.13)	0.004	−0.76 (−1.09, −0.43)	<0.001	1.04 (1.02, 1.05)	<0.001	0.74 (0.73, 0.75)	<0.001	0.86 (0.85, 0.86)	<0.001
Puerto Rico	1.10 (0.86, 1.35)	<0.001	0.27 (0.09, 0.45)	0.006	0.30 (−0.11, 0.72)	0.163	1.11 (1.10, 1.12)	<0.001	1.19 (1.18, 1.21)	<0.001	1.65 (1.62, 1.68)	<0.001
Qatar	1.61 (1.35, 1.87)	<0.001	−0.17 (−0.25, −0.09)	<0.001	−0.63 (−1.07, −0.20)	0.008	1.51 (1.49, 1.53)	<0.001	0.27 (0.27, 0.27)	<0.001	1.49 (1.47, 1.51)	<0.001
Republic of Korea	6.16 (4.49, 7.84)	<0.001	1.94 (1.45, 2.42)	<0.001	4.26 (2.90, 5.62)	<0.001	−1.17 (−1.19, −1.16)	<0.001	0.97 (0.96, 0.98)	<0.001	0.28 (0.28, 0.28)	<0.001

Republic of Moldova	1.77 (1.58, 1.97)	<0.001	0.45 (0.35, 0.56)	<0.001	1.06 (0.77, 1.36)	<0.001	−0.78 (−0.78, −0.77)	<0.001	−0.66 (−0.67, −0.66)	<0.001	−2.36 (−2.42, −2.30)	<0.001
Romania	1.56 (1.40, 1.72)	<0.001	0.32 (0.23, 0.42)	<0.001	0.17 (−0.07, 0.42)	0.173	1.34 (1.33, 1.36)	<0.001	0.33 (0.33, 0.33)	<0.001	0.64 (0.64, 0.65)	<0.001
Russian Federation	2.49 (2.23, 2.75)	<0.001	0.45 (0.29, 0.61)	<0.001	0.91 (0.54, 1.28)	<0.001	−0.25 (−0.25, −0.25)	<0.001	−0.16 (−0.16, −0.16)	<0.001	−0.64 (−0.65, −0.64)	<0.001
Rwanda	0.67 (0.48, 0.86)	<0.001	−0.12 (−0.22, −0.01)	0.033	−0.62 (−0.91, −0.33)	<0.001	1.83 (1.80, 1.87)	<0.001	0.61 (0.60, 0.61)	<0.001	1.50 (1.47, 1.52)	<0.001
Saint Kitts and Nevis	1.36 (1.11, 1.62)	<0.001	0.16 (−0.05, 0.36)	0.153	0.80 (0.35, 1.26)	0.002	3.11 (3.01, 3.21)	<0.001	2.29 (2.23, 2.35)	<0.001	3.81 (3.66, 3.97)	<0.001
Saint Lucia	1.81 (1.55, 2.06)	<0.001	0.46 (0.25, 0.68)	<0.001	1.10 (0.64, 1.56)	<0.001	1.86 (1.83, 1.90)	<0.001	1.30 (1.28, 1.32)	<0.001	2.09 (2.05, 2.14)	<0.001
Saint Vincent and the Grenadines	1.98 (1.79, 2.17)	<0.001	1.04 (0.83, 1.25)	<0.001	2.14 (1.77, 2.51)	<0.001	1.28 (1.26, 1.30)	<0.001	0.49 (0.49, 0.49)	<0.001	0.86 (0.85, 0.87)	<0.001
Samoa	0.92 (0.68, 1.16)	<0.001	0.08 (0.03, 0.12)	0.002	0.22 (0.08, 0.36)	0.005	1.84 (1.81, 1.88)	<0.001	0.06 (0.06, 0.06)	<0.001	0.37 (0.37, 0.37)	<0.001
San Marino	1.37 (1.33, 1.41)	<0.001	0.50 (0.47, 0.54)	<0.001	0.49 (0.40, 0.57)	<0.001	0.63 (0.63, 0.63)	<0.001	0.35 (0.35, 0.35)	<0.001	0.56 (0.56, 0.56)	<0.001
Sao Tome and Principe	0.64 (0.58, 0.71)	<0.001	0.10 (0.09, 0.12)	<0.001	0.79 (0.60, 0.99)	<0.001	0.96 (0.95, 0.97)	<0.001	0.14 (0.14, 0.14)	<0.001	0.74 (0.74, 0.75)	<0.001
Saudi Arabia	5.19 (4.96, 5.42)	<0.001	0.45 (0.40, 0.50)	<0.001	3.08 (2.93, 3.23)	<0.001	3.44 (3.31, 3.56)	<0.001	0.54 (0.53, 0.54)	<0.001	2.41 (2.35, 2.48)	<0.001
Senegal	0.17 (0.13, 0.21)	<0.001	0.03 (0.01, 0.05)	0.003	0.12 (−0.04, 0.27)	0.147	0.37 (0.37, 0.37)	<0.001	0.02 (0.02, 0.02)	<0.001	−0.02 (−0.02, −0.02)	<0.001

Serbia	1.83 (1.72, 1.95)	<0.001	0.65 (0.57, 0.73)	<0.001	0.84 (0.65, 1.02)	<0.001	0.14 (0.14, 0.14)	<0.001	0.23 (0.23, 0.23)	<0.001	−0.09 (−0.09, −0.09)	<0.001
Seychelles	1.02 (0.89, 1.14)	<0.001	−0.01 (−0.08, 0.05)	0.667	0.15 (−0.05, 0.36)	0.16	2.20 (2.15, 2.25)	<0.001	0.70 (0.70, 0.71)	<0.001	2.29 (2.23, 2.34)	<0.001
Sierra Leone	0.05 (0.02, 0.08)	0.004	−0.12 (−0.14, −0.10)	<0.001	−0.51 (−0.62, −0.40)	<0.001	0.46 (0.45, 0.46)	<0.001	0.03 (0.03, 0.03)	<0.001	0.48 (0.47, 0.48)	<0.001
Singapore	1.31 (1.06, 1.56)	<0.001	0.08 (0.01, 0.14)	0.023	−0.08 (−0.21, 0.05)	0.239	1.85 (1.81, 1.88)	<0.001	1.21 (1.19, 1.23)	<0.001	2.25 (2.20, 2.30)	<0.001
Slovakia	1.03 (0.95, 1.12)	<0.001	−0.04 (−0.08, −0.00)	0.054	−0.33 (−0.43, −0.23)	<0.001	1.50 (1.48, 1.52)	<0.001	0.72 (0.71, 0.72)	<0.001	1.32 (1.30, 1.34)	<0.001
Slovenia	0.92 (0.81, 1.03)	<0.001	0.13 (0.09, 0.17)	<0.001	−0.26 (−0.35, −0.16)	<0.001	1.34 (1.32, 1.35)	<0.001	0.85 (0.84, 0.86)	<0.001	1.27 (1.25, 1.28)	<0.001
Solomon Islands	1.47 (1.36, 1.57)	<0.001	0.35 (0.29, 0.41)	<0.001	1.14 (0.96, 1.32)	<0.001	1.14 (1.13, 1.15)	<0.001	0.13 (0.13, 0.13)	<0.001	0.37 (0.37, 0.37)	<0.001
Somalia	0.38 (0.32, 0.44)	<0.001	0.17 (0.14, 0.20)	<0.001	0.59 (0.51, 0.67)	<0.001	0.14 (0.13, 0.14)	<0.001	0.00 (0.00, 0.00)	<0.001	−0.00 (−0.00, −0.00)	<0.001
South Africa	0.33 (0.27, 0.39)	<0.001	0.18 (0.12, 0.24)	<0.001	0.25 (0.02, 0.49)	0.042	0.08 (0.08, 0.08)	<0.001	−0.50 (−0.50, −0.50)	<0.001	−1.76 (−1.80, −1.73)	<0.001
South Sudan	0.39 (0.30, 0.48)	<0.001	0.11 (0.05, 0.17)	0.002	0.59 (0.33, 0.85)	<0.001	0.86 (0.85, 0.87)	<0.001	0.37 (0.37, 0.37)	<0.001	1.32 (1.30, 1.34)	<0.001
Spain	1.38 (1.19, 1.56)	<0.001	0.24 (0.19, 0.29)	<0.001	0.10 (0.05, 0.16)	0.001	0.95 (0.94, 0.96)	<0.001	0.66 (0.66, 0.66)	<0.001	0.99 (0.98, 1.00)	<0.001

Sri Lanka	1.67 (1.43, 1.91)	<0.001	0.30 (0.16, 0.45)	<0.001	0.31 (−0.03, 0.66)	0.085	2.32 (2.26, 2.38)	<0.001	0.83 (0.82, 0.83)	<0.001	1.52 (1.50, 1.55)	<0.001
Sudan	1.30 (1.16, 1.44)	<0.001	0.11 (0.08, 0.15)	<0.001	0.70 (0.52, 0.89)	<0.001	2.27 (2.22, 2.33)	<0.001	0.29 (0.29, 0.29)	<0.001	1.28 (1.27, 1.30)	<0.001
Suriname	1.38 (1.14, 1.62)	<0.001	0.54 (0.41, 0.66)	<0.001	1.39 (1.06, 1.72)	<0.001	1.62 (1.59, 1.65)	<0.001	0.86 (0.85, 0.86)	<0.001	1.76 (1.73, 1.80)	<0.001
Sweden	0.63 (0.52, 0.74)	<0.001	−0.16 (−0.26, −0.07)	0.002	−0.26 (−0.46, −0.07)	0.012	−0.54 (−0.54, −0.54)	<0.001	−0.08 (−0.08, −0.08)	<0.001	−0.50 (−0.50, −0.50)	<0.001
Switzerland	1.76 (1.22, 2.31)	<0.001	0.77 (0.55, 1.00)	<0.001	1.22 (0.77, 1.67)	<0.001	0.17 (0.16, 0.17)	<0.001	−0.03 (−0.03, −0.03)	<0.001	−0.09 (−0.09, −0.09)	<0.001
Syrian Arab Republic	1.46 (1.30, 1.62)	<0.001	0.18 (0.12, 0.24)	<0.001	1.42 (0.99, 1.85)	<0.001	1.35 (1.33, 1.37)	<0.001	0.38 (0.38, 0.39)	<0.001	1.83 (1.80, 1.87)	<0.001
Taiwan (Province of China)	2.65 (2.37, 2.92)	<0.001	0.96 (0.92, 1.00)	<0.001	1.62 (1.53, 1.71)	<0.001	2.04 (1.99, 2.08)	<0.001	1.17 (1.15, 1.18)	<0.001	2.12 (2.07, 2.17)	<0.001
Tajikistan	0.18 (0.11, 0.25)	<0.001	−0.01 (−0.03, 0.01)	0.298	0.10 (−0.10, 0.30)	0.348	0.48 (0.48, 0.49)	<0.001	−0.02 (−0.02, −0.02)	<0.001	0.66 (0.65, 0.66)	<0.001
Thailand	1.39 (1.03, 1.76)	<0.001	0.53 (0.46, 0.60)	<0.001	0.25 (−0.04, 0.55)	0.098	2.59 (2.52, 2.66)	<0.001	1.57 (1.54, 1.60)	<0.001	2.53 (2.46, 2.60)	<0.001
Timor-Leste	1.50 (1.36, 1.64)	<0.001	0.72 (0.67, 0.76)	<0.001	1.38 (1.22, 1.54)	<0.001	0.81 (0.80, 0.81)	<0.001	0.24 (0.24, 0.24)	<0.001	0.53 (0.53, 0.53)	<0.001
Togo	0.21 (0.16, 0.26)	<0.001	0.05 (0.04, 0.06)	<0.001	0.26 (0.17, 0.35)	<0.001	0.76 (0.75, 0.76)	<0.001	0.17 (0.17, 0.17)	<0.001	0.95 (0.94, 0.96)	<0.001
Tokelau	1.49 (1.39, 1.59)	<0.001	0.17 (0.07, 0.27)	0.003	0.43 (0.28, 0.57)	<0.001	1.84 (1.81, 1.88)	<0.001	−0.21 (−0.21, −0.21)	<0.001	0.65 (0.64, 0.65)	<0.001
Tonga	1.02 (0.88, 1.16)	<0.001	0.44 (0.37, 0.51)	<0.001	1.10 (0.85, 1.36)	<0.001	1.42 (1.40, 1.44)	<0.001	0.47 (0.46, 0.47)	<0.001	1.08 (1.07, 1.09)	<0.001

Trinidad and Tobago	1.18 (0.96, 1.41)	<0.001	0.29 (0.16, 0.43)	<0.001	0.68 (0.32, 1.03)	<0.001	1.51 (1.49, 1.54)	<0.001	1.00 (0.99, 1.01)	<0.001	1.74 (1.71, 1.77)	<0.001
Tunisia	2.85 (2.79, 2.91)	<0.001	0.51 (0.50, 0.53)	<0.001	1.75 (1.71, 1.78)	<0.001	2.01 (1.97, 2.05)	<0.001	0.52 (0.52, 0.52)	<0.001	1.41 (1.39, 1.43)	<0.001
Turkey	2.57 (2.42, 2.71)	<0.001	0.31 (0.25, 0.37)	<0.001	0.44 (0.32, 0.56)	<0.001	2.46 (2.40, 2.52)	<0.001	0.57 (0.56, 0.57)	<0.001	1.50 (1.47, 1.52)	<0.001
Turkmenistan	1.83 (1.50, 2.16)	<0.001	0.30 (0.19, 0.41)	<0.001	1.61 (1.05, 2.17)	<0.001	0.79 (0.78, 0.80)	<0.001	0.24 (0.24, 0.24)	<0.001	0.40 (0.40, 0.40)	<0.001
Tuvalu	0.70 (0.58, 0.82)	<0.001	0.03 (−0.02, 0.08)	0.285	−0.01 (−0.10, 0.08)	0.824	1.13 (1.11, 1.14)	<0.001	0.20 (0.20, 0.21)	<0.001	0.31 (0.31, 0.31)	<0.001
Uganda	1.40 (1.35, 1.44)	<0.001	0.44 (0.43, 0.46)	<0.001	2.07 (1.91, 2.22)	<0.001	1.29 (1.28, 1.31)	<0.001	0.10 (0.10, 0.10)	<0.001	0.30 (0.30, 0.30)	<0.001
Ukraine	2.10 (1.90, 2.29)	<0.001	0.41 (0.32, 0.50)	<0.001	1.02 (0.79, 1.26)	<0.001	0.58 (0.58, 0.58)	<0.001	0.54 (0.54, 0.55)	<0.001	0.79 (0.78, 0.79)	<0.001
United Arab Emirates	2.81 (2.46, 3.16)	<0.001	0.44 (0.31, 0.57)	<0.001	2.28 (1.85, 2.70)	<0.001	4.19 (4.00, 4.37)	<0.001	1.99 (1.95, 2.03)	<0.001	4.36 (4.16, 4.56)	<0.001
UK	1.05 (0.99, 1.12)	<0.001	0.05 (−0.01, 0.10)	0.091	−0.01 (−0.12, 0.11)	0.882	0.56 (0.56, 0.57)	<0.001	0.46 (0.46, 0.46)	<0.001	0.81 (0.81, 0.82)	<0.001
United Republic of Tanzania	0.93 (0.82, 1.03)	<0.001	0.27 (0.23, 0.31)	<0.001	0.98 (0.82, 1.14)	<0.001	1.81 (1.77, 1.84)	<0.001	0.35 (0.35, 0.35)	<0.001	1.24 (1.22, 1.25)	<0.001
United States Virgin Islands	2.45 (2.24, 2.66)	<0.001	1.47 (1.34, 1.60)	<0.001	3.09 (2.83, 3.36)	<0.001	−0.21 (−0.21, −0.21)	<0.001	0.55 (0.55, 0.55)	<0.001	0.21 (0.21, 0.21)	<0.001
USA	1.65 (1.52, 1.77)	<0.001	0.54 (0.52, 0.56)	<0.001	1.25 (1.21, 1.28)	<0.001	0.23 (0.23, 0.23)	<0.001	0.66 (0.65, 0.66)	<0.001	0.84 (0.83, 0.84)	<0.001
Uruguay	1.70 (1.36, 2.05)	<0.001	0.57 (0.40, 0.74)	<0.001	1.12 (0.72, 1.53)	<0.001	1.37 (1.35, 1.39)	<0.001	0.47 (0.47, 0.47)	<0.001	0.88 (0.87, 0.88)	<0.001
Uzbekistan	1.21 (1.07, 1.34)	<0.001	0.23 (0.20, 0.26)	<0.001	2.07 (1.86, 2.27)	<0.001	0.73 (0.73, 0.74)	<0.001	0.00 (0.00, 0.00)	<0.001	0.20 (0.20, 0.20)	<0.001

Vanuatu	0.94 (0.84, 1.03)	<0.001	0.44 (0.40, 0.49)	<0.001	1.40 (1.26, 1.53)	<0.001	0.84 (0.83, 0.85)	<0.001	0.35 (0.35, 0.35)	<0.001	0.68 (0.67, 0.68)	<0.001
Venezuela (Bolivarian Republic of)	2.52 (2.43, 2.61)	<0.001	0.82 (0.75, 0.89)	<0.001	2.44 (2.31, 2.57)	<0.001	2.29 (2.23, 2.34)	<0.001	2.06 (2.01, 2.10)	<0.001	3.63 (3.50, 3.77)	<0.001
Viet Nam	5.14 (4.72, 5.55)	<0.001	1.65 (1.47, 1.84)	<0.001	3.81 (3.35, 4.27)	<0.001	2.56 (2.50, 2.63)	<0.001	0.89 (0.89, 0.90)	<0.001	1.42 (1.40, 1.44)	<0.001
Yemen	1.38 (1.27, 1.48)	<0.001	0.19 (0.16, 0.21)	<0.001	1.14 (1.00, 1.28)	<0.001	1.29 (1.27, 1.31)	<0.001	0.43 (0.43, 0.43)	<0.001	1.99 (1.94, 2.03)	<0.001
Zambia	1.03 (0.91, 1.15)	<0.001	0.15 (0.14, 0.16)	<0.001	0.53 (0.48, 0.59)	<0.001	1.90 (1.86, 1.94)	<0.001	0.34 (0.34, 0.34)	<0.001	0.99 (0.98, 1.00)	<0.001
Zimbabwe	1.07 (0.95, 1.20)	<0.001	0.61 (0.53, 0.69)	<0.001	2.39 (2.06, 2.72)	<0.001	1.20 (1.19, 1.22)	<0.001	0.23 (0.23, 0.23)	<0.001	0.75 (0.74, 0.75)	<0.001

ASDR − age-standardised death rate, ASIR − age-standardised incidence rate, CI – confidence interval, DALY − disability-adjusted life years, EAPC – estimated annual percentage changes

**t*-test.
